# Tandem Catalysis in CuO/ZnMnO_3_ Heterostructures for Efficient Electrooxidation of N_2_ to Nitrate

**DOI:** 10.1021/jacs.6c10955

**Published:** 2026-08-26

**Authors:** Yuan Yuan, Xitang Qian, Shiyuan Liu, Qingbo Wa, Yinuo Wang, Xiaoyi Qiu, Yan Zhang, Siqi Lu, Yuxiang Lyu, Hua Zhang, Minhua Shao

**Affiliations:** † Department of Chemical and Biological Engineering, 58207 The Hong Kong University of Science and Technology, Clear Water Bay, Kowloon, Hong Kong 999077, China; ‡ Department of Chemistry, 53025 City University of Hong Kong, Hong Kong 999077, China; § CIAC-HKUST Joint Laboratory for Hydrogen Energy, The Hong Kong University of Science and Technology, Clear Water Bay, Kowloon, Hong Kong 999077, China; ∥ Fok Ying Tung Research Institute, The Hong Kong University of Science and Technology, Guangzhou 511458, China

## Abstract

Nitrate is indispensable in modern agriculture and industry, yet its production via the energy-intensive Haber-Bosch and Ostwald oxidation processes results in significant carbon emissions. Electrocatalytic nitrogen oxidation reaction (eNOR) offers a highly sustainable and decentralized alternative for nitrate synthesis. However, the inherent challenge of N_2_ activation, stemming from its extremely inert triple bond, and the kinetically sluggish nature of the multielectron-transfer pathway severely impede its practical application. Inspired by multielectron cascade processes, we rationally engineered a CuO/ZnMnO_3_ heterostructure catalyst to leverage tandem catalysis, deconstructing the formidable multielectron-transfer pathway of N_2_ oxidation. This innovative heterostructure spatially decouples nitrogen activation from subsequent oxidation, effectively breaking down the overall reaction into lower-energy, stepwise transformations. The catalyst achieves an enhanced nitrate yield rate of 52.1 μmol h^–1^ mg^–1^ and a Faradaic efficiency of 45.2%, surpassing single-component benchmarks (1.6-fold higher activity than ZnMnO_3_) and most reported state-of-the-art eNOR catalysts with exceptional long-term stability. Through comprehensive kinetic analysis, in situ differential electrochemical mass spectrometry, and density functional theory calculations, we provide compelling evidence supporting the tandem reaction mechanism. Specifically, N_2_ is initially activated and partially oxidized to the key intermediate N_2_O on the ZnMnO_3_ component, which is then efficiently captured and further oxidized to nitrate on the CuO component. This work not only presents a highly efficient and stable catalyst for sustainable nitrate electrosynthesis but also validates tandem-site engineering as a strategic paradigm for managing complex multielectron transfer reactions.

## Graphical abstract

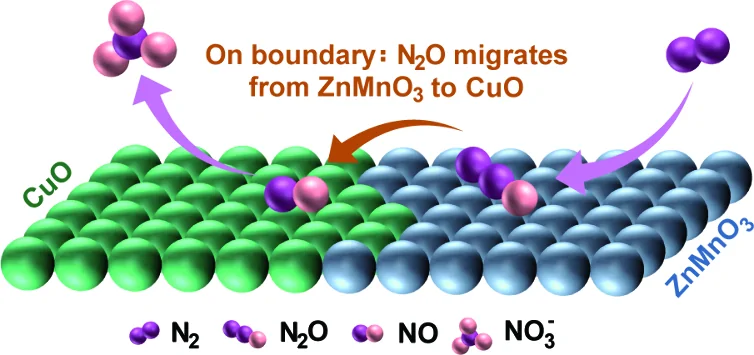

## Introduction

Nitrates are indispensable precursors for fertilizers, explosives, and fine chemicals, underpinning global food security and industrial productivity.^1,2^ Contemporary nitrate production relies exclusively on the century-old Haber–Bosch (HB) process for ammonia synthesis, followed by Ostwald oxidation.^3,4^ Collectively, these processes consume ∼2% of worldwide energy and emit over 400 million tons of CO_2_ annually.^5,6^ This energy-intensive paradigm, coupled with the requirement for centralized infrastructure, imposes severe environmental and logistical constraints. The electrocatalytic nitrogen oxidation reaction (eNOR), which directly converts N_2_ to nitrate (N_2_ + 12OH^–^ → 2NO_3_^–^ + 6H_2_O + 10e^–^) under ambient conditions using renewable electricity, has emerged as a promising sustainable alternative.^7,8^ Unlike thermochemical processes, eNOR eliminates direct carbon emissions and enables decentralized production, aligning with green chemistry principles. However, eNOR faces formidable mechanistic challenges. The extreme stability of the N≡N bond (bond dissociation energy: 9.7 eV) necessitates high activation barriers, while the reaction involves a complex 10-electron transfer process with multiple intermediates (e.g., N_2_O and NO) and divergent kinetic pathways.^3^ Therefore, an ideal catalyst must be designed to simultaneously facilitate N_2_ activation and intermediate stabilization, thereby effectively bypassing kinetic traps and promoting deep oxidation. Transition metals address these needs through orbital interactions, where σ-donation from the highest occupied molecular orbital (HOMO) of N_2_ to vacant metal d-orbitals lowers the energy barrier and π-backdonation from filled metal d-orbitals to the lowest unoccupied molecular orbital (LUMO) of N_2_ weakens the N≡N bond.^9^ Given that the adsorption energy of distinct intermediates varies significantly across different active sites, the integration of complementary components via a tandem mechanism allows for the decoupling of energetically demanding steps. Consequently, constructing heterostructured catalysts that enable one site to activate N_2_ into a reactive intermediate and the other site to perform further oxidation represents a promising strategy to lower the overall overpotential.^10^

Consequently, we engineer a CuO/ZnMnO_3_ heterostructure catalyst for efficient eNOR tandem catalysis. As reported, Zn-based oxides (e.g., ZnFe_2_O_4_, ZnCo_2_O_4_, La-ZnO) have been reported to exhibit favorable N_2_ adsorption behavior, which can be associated with Lewis-acidic Zn^2+^ centers and defect-modulated local electronic structures.^11,12^ These features can promote the interaction between N_2_ and the oxide surface while suppressing competing OER through unfavorable *OH binding.^12–14^ Incorporating Mn further enhances N_2_ activation via the empty d orbitals of interfacial Mn atoms.^15^ For the secondary intermediate oxidation site, CuO demonstrates superior activity toward nitrous oxide (N_2_O) decomposition and oxidation due to its moderate affinity for nitrogen oxides and variable valence states.^10^ We thus construct a heterostructure in which the ZnMnO_3_ phase activates N_2_ to N_2_O, and the adjacent CuO phase catalyzes N_2_O to nitrate. This catalyst achieves a nitrate yield rate of 52.1 μmol h^–1^ mg^–1^ and a Faradaic efficiency (FE) of 45.2%, significantly outperforming single-component benchmarks (1.6 times higher activity than ZnMnO_3_).^9,12,16–27^ Through detailed kinetics analysis, in situ differential electrochemical mass spectrometry (DEMS), and density functional theory (DFT) calculations, we propose the tandem reaction mechanism with N_2_O as intermediates. Specifically, ZnMnO_3_ adsorbs and oxidizes N_2_ to N_2_O, while CuO selectively converts N_2_O to nitrate, collectively bypassing the single-site scaling relations that limit conventional eNOR catalysts.

## Experimental Section

### Materials

Zinc acetate hexahydrate (Zn­(CO_2_CH_3_)_2_·6H_2_O) (99%), Copper acetate monohydrate (Cu­(CO_2_CH_3_)_2_·H_2_O) (99%), Manganese acetate tetrahydrate (Mn­(CO_2_CH_3_)_2_·4H_2_O) (99%), citric acid (HOC­(COOH)­(CH_2_COOH)_2_) (99%) and potassium hydroxide (KOH) (99.99%) were obtained from Aladdin. Hydrochloride (HCl) (37%) and sulfuric acid (H_2_SO_4_) (96%) were obtained from Honeywell Fluka. Nitric acid (HNO_3_) (70%) was obtained from VWR Chemicals. Carbon Cloth (CC) was obtained from Taiwan CeTech Co., Ltd. All chemicals were used as received. All solutions were prepared using deionized (DI) Milli-Q water (15 MΩ cm^–1^ at 25 °C).

### Synthesis of CuO, ZnMnO_3_, and CuO/ZnMnO_3_

Heterogeneous CuO/ZnMnO_3_ materials were synthesized by a sol–gel combustion method. Stoichiometric amounts of Cu­(CO_2_CH_3_)_2_·H_2_O, Zn­(CO_2_CH_3_)_2_·6H_2_O, and Mn­(CO_2_CH_3_)_2_·4H_2_O were first mixed and dissolved in the solution containing 15 mL of double-distilled water and 3 mmol citric acid) under magnetic stirring following the molar ratio of 1:1:1 (1 mmol:1 mmol:1 mmol). The mixture solution was converted into a viscous gel after constant stirring at 90 °C for 3 h. The obtained gel was then decomposed in air at 500 °C with a heating rate of 5 °C min^–1^ for 5 h to remove the remaining water, and later ground thoroughly into powder. Then, the powder was heat-treated in air at 700 °C with a heating rate of 10 °C min^–1^ for 4 h. ZnMnO_3_ and CuO followed identical protocols, without heterometallic precursors. 0.5CuO/ZnMnO_3_ heterostructures were synthesized using 0.5 mmol Cu­(CO_2_CH_3_)_2_·H_2_O, 1.5 mmol Zn­(CO_2_CH_3_)_2_·6H_2_O, 1.5 mmol Mn­(CO_2_CH_3_)_2_·4H_2_O, and 3.5 mmol citric acid as raw materials. 1.5CuO/ZnMnO_3_ heterostructures were synthesized using 1.5 mmol Cu­(CO_2_CH_3_)_2_·H_2_O, 0.5 mmol Zn­(CO_2_CH_3_)_2_·6H_2_O, 0.5 mmol Mn­(CO_2_CH_3_)_2_·4H_2_O, and 2.5 mmol citric acid as raw materials.

### Material Characterizations

Scanning Electron Microscopy (SEM) mapping was carried out on a JEOL JSM-6700F. Aberration-corrected Scanning Transmission Electron Microscopy (STEM) was carried out on a JEOL JEM-ARM200F at an accelerating voltage of 200 kV. X-ray diffraction (XRD) patterns of the catalysts were obtained by using Panalytical X’pert Pro. X-ray Photoelectron Spectroscopy (XPS) measurements were performed on an Axis Ultra DLD. The C 1s peak at 284.4 eV was used to correct the binding energies of all the catalysts. UV–vis absorption spectra were recorded on an Agilent UV–vis spectrophotometer. Ion-chromatography (IC) was performed on a Metrohm/881 IC. The amount of metal elements was detected by Inductively Coupled Plasma Mass Spectrometry (ICP-MS) by using PwekinElmer/7300DV after the microwave-digestion process (Milestone/Ethos One). The Raman spectra were recorded by Renishaw inVia Qontor Spectrometer System use a 785 nm laser. The electron paramagnetic resonance (EPR) spectra were measured by using an EPR spectrometer (Bruker A300) at 9.85 GHz. The N_2_ temperature-programmed desorption (N_2_-TPD) measurements were conducted by Chemisorption instrument (AMI-300, Altamira, USA). The catalyst was placed at the bottom of the tube and heated from room temperature to 300 °C at a rate of 10 °C min^–1^ and kept for 1 h with a gas flow of He (50 mL min^–1^). After it was cooled down to 50 °C, the gas flow switched to N_2_ (50 mL min^–1^) and kept for another 1 h to ensure saturated adsorption. Afterward, the gas flow was then switched back to He (50 mL min^–1^) and kept for 1 h to remove the physically adsorbed N_2_ at the catalyst surface. Finally, it was desorbed from 50 to 700 °C at a heating rate of 10 °C min^–1^ in a gas flow of He (50 mL min^–1^).

### Electrochemical Measurement

Before the electrochemical tests, the blown N_2_ (99.9999% purity) and Ar (99.99% purity) were prepurified by passing through an alkaline trap with 0.1 M KMnO_4_ dissolved in 0.1 M KOH followed by an acidic trap with 0.1 M H_2_SO_4_ solution to remove any possible impurities containing N-contaminations before feeding into the electrolyte. Electrochemical nitrogen oxidation reaction (eNOR) tests were carried out in a gastight H-type cell (50 mL in volume) separated by a membrane (Nafion 117). Before NOR tests, the membrane was heat-treated in 5 wt % H_2_O_2_, 0.5 M H_2_SO_4_, and ultrapure water at 80 °C for 1 h, respectively. After rinsing thoroughly with water, the membrane is immersed in deionized water for later use. In a typical case, the catalyst ink was prepared through ultrasonic treatment of a suspension containing catalysts (3 mg), isopropanol (250 μL), DDI water (750 μL), and 5 wt % Nafion solution (50 μL). The working electrode was prepared by injecting 12.5 μL catalyst ink on CC with a fixed area of 0.5 × 0.5 cm^2^. The electrochemical tests were carried out on a Gamry Reference 3000 electrochemical station. The electrochemical workstation uses a three-electrode configuration including a working electrode (carbon cloth of 0.25 cm^2^ of catalyst@CC), reference electrode (Hg/HgO electrode), counter electrode (Pt mesh), and electrolyte (1 M KOH solution). All electrochemical tests were carried out at ambient conditions. To calibrate the reference electrode against the RHE, an alkaline electrolyte was first prepared by dissolving KOH to obtain a solution with a pH of 13. Prior to the measurement, high-purity H_2_ gas was introduced into the electrolyte at a constant flow rate of 20 mL min^–1^ for 30 min to ensure a saturated hydrogen environment. A clean Pt wire was used as the working electrode and immersed in the H_2_-saturated electrolyte. The open-circuit potential (E_OCP_) of the Pt wire versus the reference electrode was then recorded. Under these conditions, the measured E_OCP_ corresponds to the potential difference between the reference electrode and the RHE. Accordingly, all potentials reported in this work were converted to the RHE scale using E_RHE_ = E_measured_ + E_OCP_. For alkaline media with known pH of 14, the equation can also be written as E_RHE_ = E_measured_ + E_OCP_ + 0.059. For eNOR experiments, N_2_ was blown into the anode chamber at a flow rate of 50 mL min^–1^. The electrolyte was purged with N_2_ or Ar for 30 min to remove the residual air gas and make the N_2_ saturated. Before all electrochemical tests, several linear sweep voltammetry (LSV) tests were performed with a scan rate of 100 mV s^–1^ between the open circuit potential (OCP) and 2.4 V vs RHE to reach the steady state. The chronoamperometry (CA) tests were carried out at a series of applied potentials for 2 h. For the consecutive cycling repetitive NOR test, independent repetitive eNOR experiment was conducted for 3 times using the above-mentioned process of eNOR test. The potentials were converted to the RHE scale by calibration with a reversible hydrogen electrode in 0.1 M KOH electrolyte.

Then, RRDE experiments were applied to capture intermediates during the eNOR process. A preliminary electrochemical characterization was performed to evaluate the sensitivity of the Pt ring and its surface electrochemistry, as various products can be generated simultaneously on the glassy carbon (GC) disk surface coated with eNOR electroactive materials at high positive potentials (e.g., NO_3_^–^, NO, and/or NO_2_^–^). CVs on the Pt electrode were conducted in an Ar-purified 1 M KOH electrolyte solution and with the addition of 0.1 M KNO_2_ within the water stability window (0–1.4 V vs RHE). The Pt ring exhibited high activity toward nitrite reduction, as indicated by an obvious reduction peak at 0.12 V vs RHE, as shown in our previous work.^11^ A preliminary scan of the relevant catalytic potentials was performed using CA on the covered disk electrode at 2.1 V vs RHE, with the ring potential scanned within the water stability window.

In situ Raman measurements were performed using the same Raman spectrometer equipped with an electrochemical Raman cell. The catalyst-loaded carbon cloth was used as the working electrode, with a Hg/HgO electrode and a Pt mesh as the reference and counter electrodes, respectively. The electrolyte was 1 M KOH. Before Raman acquisition, the electrolyte was purged with high-purity N_2_ to ensure N_2_-saturated conditions. Time-dependent Raman spectra were collected at a constant potential of 2.1 V vs RHE for 270 min, corresponding to the potential at which the highest nitrate Faradaic efficiency was obtained. The spectral evolution was used to monitor the surface structural changes of CuO/ZnMnO_3_ under anodic eNOR conditions.

For in situ DEMS experiments, 1 M KOH solution was kept continuously flowing into a handmade electrochemical cell at a roughly constant flow rate. Prior to and throughout the DEMS measurements, Ar or N_2_ was continuously bubbled into the electrolyte. The working electrode, counter electrode, and reference electrode were CC coated with the CuO/ZnMnO_3_ catalyst, Pt mesh, and Hg/HgO electrode, respectively. There was a breathable and hydrophobic membrane without a Nafion membrane in the experimental setup. The distance between the vacuum chamber of mass spectrometry and the surface of the working electrode was fixed, which was basically consistent in each experiment. After the baseline was maintained, it was employed from 1.0 to 2.2 V vs RHE. Then, the accompanying mass signals began to appear. Once the electrochemical test was finished and the mass signal returned to baseline, the subsequent cycle began under identical conditions to prevent unintentional errors. The experiment was completed after several cycles.

### Determination of NO_3_^–^

Ultraviolet–visible (UV–vis) spectrophotometer was employed to detect the ion concentration of the pretest and post-test electrolytes after diluting to appropriate concentration to match the range of calibration curves. Typically, an appropriate amount of electrolyte was taken out from the anode chamber and diluted into appropriate concentration. Then, 0.1 mL of 1 M HCl and 0.01 mL of 0.8 wt % H_3_NO_3_S solution were added to the aforementioned electrolyte. After sonicating for 10 min and standing for 5 min, the absorbance of NO_3_^–^ was measured by using a UV–vis spectrophotometer at a wavelength range from 300 to 200 nm. The final absorbance (A) of NO_3_^–^ was calculated based on the equation: A = A_220 nm_ – 2A_275 nm_.^26^ The standard calibration curve was established by the absorbance of the different given concentrations of KNO_3_ (Figure S9). Besides, the content of nitrate in the electrolyte was also measured by an ion chromatography (IC) workstation with anion chromatographic columns (Figure S9). Both nitrate and nitrite ions can be detected by IC tests; however, no nitrite ion was found in all product electrolytes.

### Calculation of the NO_3_^–^ Yield and Faradaic Efficiency

FEs and area-normalized yield rates of NO_3_^–^ were calculated:

$$\begin{array}{l} \mathrm{FE}\;\left({{\mathrm{NO}}_{3}}^{-}\right)=\left(5F\times C\times V\right)/Q\\ \mathrm{Yield rate}\;\left({{\mathrm{NO}}_{3}}^{-}\right)=\left(C\times V\right)/\left(t\times A\right) \end{array}$$

where F is the Faraday constant (96485 C mol^–1^), C, V, Q, and A are the measured NO_3_^–^ concentration, the volume of electrolyte, the total charge passed through the electrode, and the geometric area of the working electrode (0.25 cm^2^).

### DFT Calculation Methods

Density functional theory (DFT) calculations were performed using the Vienna Ab Initio Simulation Package (VASP).^28,29^ The interactions between valence electrons and ion cores were modeled using projector augmented wave (PAW) based potentials.^30^ The electron exchange and correlation energy were described using the generalized gradient approximation parametrized by Perdew–Burke–Ernzerhof (PBE). A plane-wave kinetic energy cutoff of 500 eV was employed. The Brillouin zone was sampled using a Monkhorst–Pack 3 × 3 × 1 k-point grid. The bottom two layers of the slab are fixed at the bulk lattice constant while the rest are allowed to relax. The geometries were optimized until the energy was converged to 1.0 × 10^–5^ eV atom^–1^, with atomic configurations relaxed to have residual forces smaller than 0.01 eV Å^–1^. The semiempirical DFT-D3 correction was applied using Grimme’s scheme.^31^ A vacuum slab of 15 Å was inserted in the *z*-direction to isolate the surfaces and avoid interactions between neighboring surfaces in all calculations.

Ab initio molecular dynamics (AIMD) simulations were conducted in a constant-volume, constant-temperature ensemble for a duration of 200 fs (fs) with a time step set to 1 fs. The simulation was aimed at mimicking the spontaneous process of N_2_O transferring from ZnMnO_3_ to CuO. The Nosé–Hoover thermostat method was employed to maintain the temperature at 298 K, which is consistent with the experimental conditions. The resulting full-precision trajectory was recorded, and visual molecular dynamics (VMD) software^32^ was used for visualization the atomic configurations.

## Results and Discussion

### Characterization of CuO/ZnMnO_3_ Heterostructure Catalyst

Scanning electron microscopy (SEM) images reveal that the synthesized CuO/ZnMnO_3_ consists of particles approximately 100 nm in diameter (Figure S1). X-ray diffraction (XRD) patterns show that the diffraction peaks of CuO/ZnMnO_3_ correspond well to monoclinic CuO (PDF#45-0937) and cubic ZnMnO_3_ (PDF#19-1461) without detectable impurity phases, supporting the immiscibility between the two components (Figures 1a and S2).^33^ Characteristic peaks at 2θ = 35.5°, 38.7°, and 48.7° are identified in the XRD patterns of CuO, which can be ascribed to (002), (111) and (−202) facets, respectively. Characteristic peaks at 2θ = 30.2, 35.6, 37.2, 43.2, 53.7, and 57.2 are identified in XRD patterns of ZnMnO_3_, which can be ascribed to (220), (311), (222), (400), (422), and (511) facets, respectively. Furthermore, the peak at the ZnMnO_3_ (220) facet of the CuO/ZnMnO_3_ (30.2°) shifts to higher angles compared to pure ZnMnO_3_ (30.1°), indicating a reduced interplanar spacing from 0.297 to 0.295 nm of ZnMnO_3_ in the heterostructure (Figure 1a inset).^34^ This contraction arises from interfacial electron transfer from ZnMnO_3_ to CuO, which optimizes the electronic structure of active sites and accelerates charge transport, thereby enhancing electrocatalytic nitrogen oxidation activity.^33^ Scanning transmission electron microscopy (STEM) was used to examine the fine structure. The elemental mapping supports the homogeneous distribution of Zn, Mn, Cu, and O within the particles (Figure 1b). Inductively coupled plasma mass spectrometry (ICP-MS) supported that the metal ratios matched those of the precursors (Table S1). As shown in Figure 1c, high-resolution TEM (HRTEM) images of CuO/ZnMnO_3_ show distinct lattice fringes in different regions. The interface between CuO and ZnMnO_3_ exhibits fringe spacings of 0.253 and 0.295 nm, corresponding to the (002) facet of CuO and the (220) facet of ZnMnO_3_, respectively. In conjunction with the XRD results, we synthesized a CuO/ZnMnO_3_ heterostructure. The tight interfacial contact ensured a physical channel for electron transfer via the bridging oxygen ions, reducing the interfacial barrier and benefiting the intermediate migration.^35^

**Figure 1:**
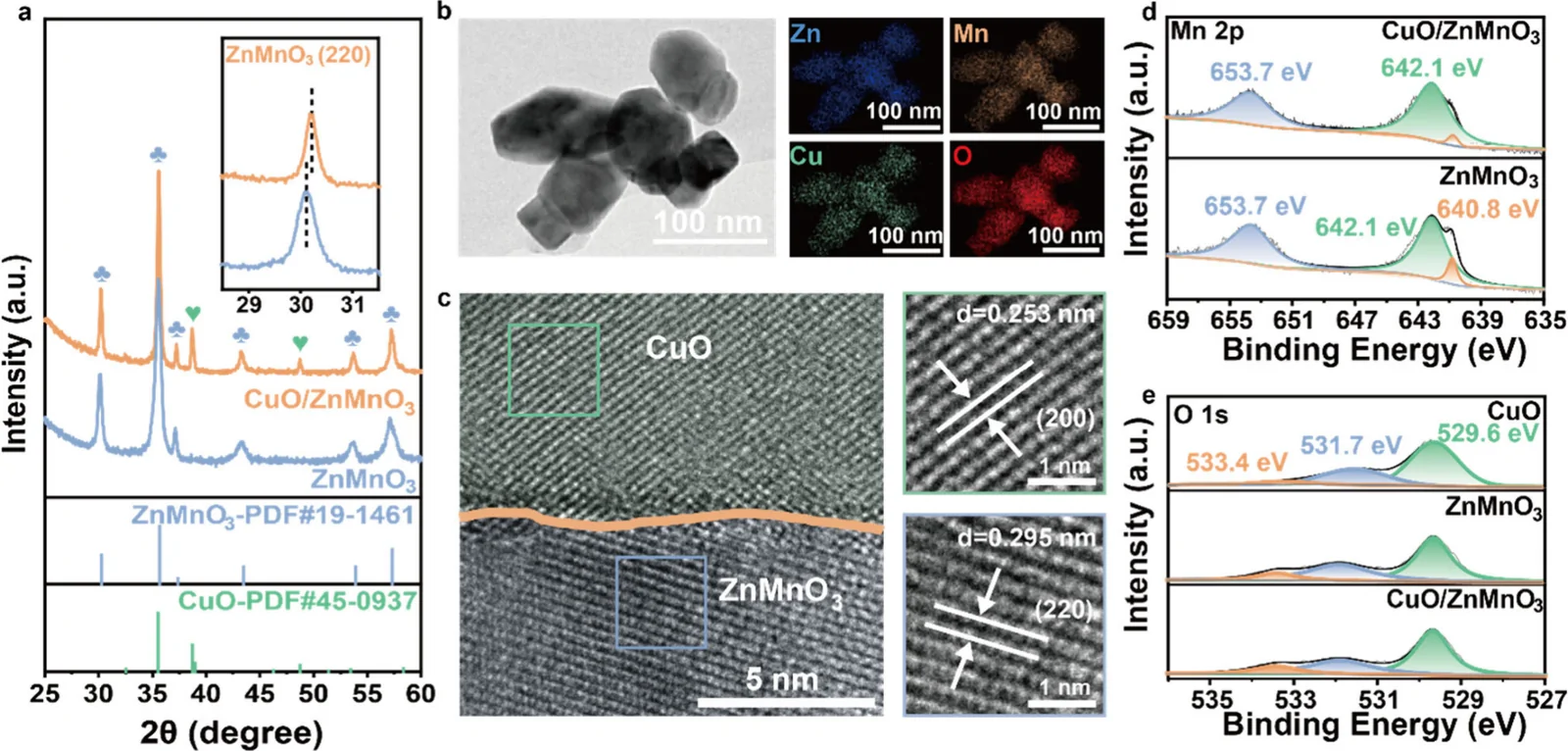
Physical and chemical characterization of CuO/ZnMnO_3_ heterostructure catalyst. (a) XRD patterns of ZnMnO_3_ and CuO/ZnMnO_3_. (b) TEM image and corresponding EDX mapping of CuO/ZnMnO_3_. (c) HRTEM image of the interface of CuO/ZnMnO_3_. (d, e) Mn 2p (d) and O 1s (e) XPS spectra of CuO, ZnMnO_3_ and CuO/ZnMnO_3_.

X-ray photoelectron spectroscopy (XPS) was then used to investigate the chemical states of CuO/ZnMnO_3_ (Tables S2–S4). The high-resolution Cu 2p spectrum (Figure S3) shows characteristic peaks at 933.9 and 953.8 eV, assigned to Cu 2p_3/2_ and Cu 2p_1/2_, respectively, along with satellite peaks indicative of Cu^II^.^36,37^ The Zn 2p spectrum exhibits peaks at 1021.1 and 1044.1 eV, supporting the Zn^II^ oxidation state (Figure S4).^37^ For pure ZnMnO_3_, the Mn 2p spectrum shows peaks at ∼642.1 eV (Mn 2p_3/2_) and ∼653.7 eV (Mn 2p_1/2_), attributing to Mn^IV^ (Figure 1d).^36^ Besides, ZnMnO_3_ shows an larger peak at 640.8 eV, which is attributed to the formation of oxygen vacancies caused by combustion during synthesis, resulting in the presence of Mn^II^.^36^ With the addition of CuO, the transfer of the electrons from Mn to Cu increases the binding energy of Mn, resulting in a higher Mn oxidation state and the decrease of the Mn^II^ peak. Mn sites modulate the local electronic structure and provide additional orbital interactions for N_2_ activation.^38^ The O 1s spectrum (Figure 1e) can be deconvoluted into three components, which are lattice oxygen (O_latt_) at ∼529.6 eV, oxygen vacancies (O_v_) at ∼531.7 eV, and surface oxygen species (O_sur_) such as hydroxyl groups or water at ∼533.4 eV.^18^ The presence of O_v_ is attributed to the combustion process of the sol–gel synthesis method and the synergistic interaction between CuO and ZnMnO_3_, which is consistent with the electron paramagnetic resonance (EPR) results (Figure S5). In addition, O_v_ is supposed to play an important role in both N_2_ activation and OER suppression, which indicates the successful fabrication of CuO/ZnMnO_3_ with modulated active sites.^18,39^ To sum up, the synergistic effect of ZnMnO_3_ and CuO forms a dual-active-site system, with ZnMnO_3_ responsible for N_2_ activation and CuO for electron transfer, respectively, further reducing the activation energy of the overall nitrogen oxidation reaction.

### Performance of the Electroconversion of N_2_ to Nitrate

The eNOR performance of the CuO/ZnMnO_3_ was evaluated in an H-type cell under ambient conditions with strict pretreatment to avoid nitrogen contamination (Figure S6). Compared with Ar-saturated baselines, linear sweep voltammetry (LSV) from 1.0 V vs RHE (Potentials are vs RHE, except where indicated) to 2.2 V in 1 M KOH under N_2_-saturated conditions shows extra current densities, indicating the nitrogen oxidation activity of CuO/ZnMnO_3_(Figure 2a).^12^ The eNOR performance on different catalysts was then assessed between 1.9 and 2.2 V in a gastight H-cell with continuous N_2_ purging. Nitrate yield rates were validated by cross-referenced UV–vis spectrophotometry and ion chromatography (IC) (Figures S7 and S8). With the applied voltage increasing, nitrate yield rates increased progressively for all catalysts. At the same applied voltage, CuO/ZnMnO_3_ shows much higher nitrate yield rates than ZnMnO_3_ and CuO. The heterostructure achieved a nitrate production rate of 52.1 μmol h^–1^ mg^–1^ at 2.2 V, which is 1.6 and 4.6 times higher than pure ZnMnO_3_ (32.7 μmol h^–1^ mg^–1^) and pure CuO (11.3 μmol h^–1^ mg^–1^) (Figures 2b, S9, and S10). FEs exhibited volcano-shaped behavior, increasing from 12.3% at 1.9 V to a maximum of 45.2% at 2.1 V, then decreasing to 12.0% at 2.2 V due to competing OER (Figure 2c). The optimal FE of 45.2% for CuO/ZnMnO_3_ catalyst shows a 25.4% improvement over ZnMnO_3_ (16.7%), demonstrating the effectiveness of the dual sites. The tandem design was validated through a series of control experiments. Pure CuO shows a negligible nitrate yield rate and eNOR FE (11.3 μmol h^–1^ mg^–1^ of the nitrate yield rate at 2.2 V and 12.0% of FE at 2.1 V) due to poor N_2_ activation, while ZnMnO_3_ achieved moderate activity (32.7 μmol h^–1^ mg^–1^ of the nitrate yield rate at 2.2 V and 16.7% of FE at 2.1 V). The superior performance of CuO/ZnMnO_3_ highlights the synergistic interfacial effect in suppressing OER and facilitating stepwise oxidation. In addition to CuO/ZnMnO_3_ with the ratio of 1:1, we have further prepared CuO/ZnMnO_3_ with the ratio of 1:3 and 3:1, and then carried out its structural characterization and eNOR performance measurements (Figures S11–S13). Compared with different ratios of CuO and ZnMnO_3_, CuO/ZnMnO_3_ with the ratio of 1:1 shows highest nitrate yield rates and FEs, which is likely attributed to the balanced active sites for N_2_ oxidation to N_2_O and N_2_O oxidation to nitrate. To further validate the synergistic interfacial effect in the heterostructure, the physically mixed CuO and ZnMnO_3_ was applied. The significantly decreased yield rates and FEs support that beyond pure spatial decoupling, interfacial synergistic effects, where the modulated local electronic structure cooperatively stabilizes key intermediates, may also play a critical concurrent role in facilitating the overall eNOR kinetics (Figure S14). Systematic control experiments ruled out artifacts. There is no detectable nitrate under N_2_ flow at open-circuit potential, supporting the absence of contamination from the inlet gas. There is also no nitrate yield under Ar flow at 2.1 V, excluding electrode/electrolyte-derived nitrogen. The pristine carbon cloth electrodes produced minimal nitrate (0.2 μmol h^–1^ mg^–1^), supporting that the eNOR activity arises from the catalyst (Figures 2d and S15). Isotopic labeling with ^15^N_2_ resulted in exclusive ^15^NO_3_^–^ formation (*m*/*z* = 63), as detected by time-of-flight secondary ion mass spectrometry (ToF-SIMS), supporting the electrocatalytic origin of nitrate from N_2_ (Figure 2e). Taking into account the variations in testing conditions across literature (such as electrolyte concentration and cell configuration), the catalyst’s performance remains highly competitive among reported state-of-the-art eNOR systems (Figure 2f and Table S5).^9,12,16–27^

**Figure 2:**
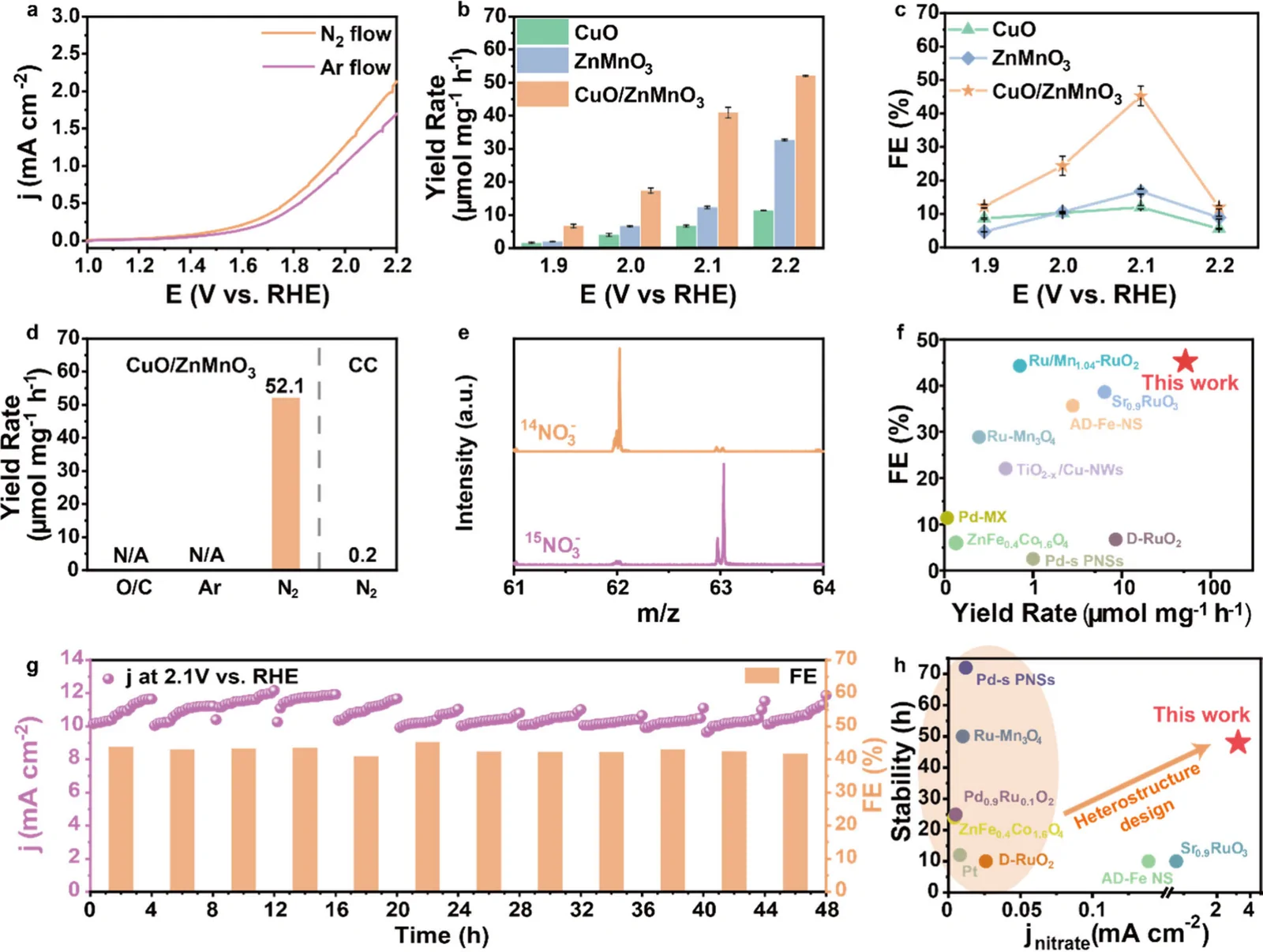
ENOR performance of CuO/ZnMnO_3_ heterostructure catalyst. (a) LSV curve of CuO/ZnMnO_3_ under Ar and N_2_ flow. (b, c) Nitrate yield rates (b) and FE (c) of CuO, ZnMnO_3_, and CuO/ZnMnO_3_ at different potentials. (d) Nitrate yield rates of CuO/ZnMnO_3_ electrode under different conditions (“Ar” indicates Ar-saturated electrolyte) and of the CC electrode in N_2_-saturated electrolyte. (e) ToF-SIMS spectra of ^14^NO_3_^–^ and ^15^NO_3_^–^ derived from the eNOR using ^14^N_2_ and ^15^N_2_ as the feeding gas. (f) Comparison of nitrate yield rates and FE values for CuO/ZnMnO_3_ with other reported eNOR catalysts. (g) Long-term stability of CuO/ZnMnO_3_ in 1 M KOH electrolyte at 2.1 V vs RHE. (h) Comparison of nitrate partial current density and stability for CuO/ZnMnO_3_ with other reported eNOR catalysts.

To verify the durability of CuO/ZnMnO_3_, stability tests were conducted at 2.1 V. The nitrate production remained stable at 40.9 μmol h^–1^ mg^–1^ and 45.2% FE over 48 h (4 h per cycle) with less than 5% decay (Figure 2g). The heterostructure catalyst combines high activity with high stability, surpassing the reported performance of conventional catalysts (Figure 2h). This combination of high activity, selectivity, and durability establishes CuO/ZnMnO_3_ as a promising catalyst for eNOR and demonstrates the effectiveness of tandem-site optimizatiion.

### Elucidating the Tandem Catalytic Mechanism on CuO/ZnMnO_3_ Catalyst

Comprehensive electrochemical analyses were conducted to elucidate the distinct roles of each component and to decipher the tandem mechanism. First, LSV measurements were performed in Ar- and N_2_-saturated 1 M KOH electrolyte (Figures 3a and S16). At 2.1 V vs RHE (potential with the highest eNOR FE), CuO shows lower current density under N_2_ atmosphere than that under Ar atmosphere (Δj = −0.36 mA cm^–2^), indicating its limited capability for initial N_2_ activation. In contrast, ZnMnO_3_ shows higher current density under N_2_ atmosphere (Δj = 0.76 mA cm^–2^), indicating its activity toward N_2_. Combined with both components, the heterostructure shows moderately higher current density under N_2_ atmosphere (Δj = 0.33 mA cm^–2^), indicating its N_2_ adsorption ability from ZnMnO_3_ component.

**Figure 3:**
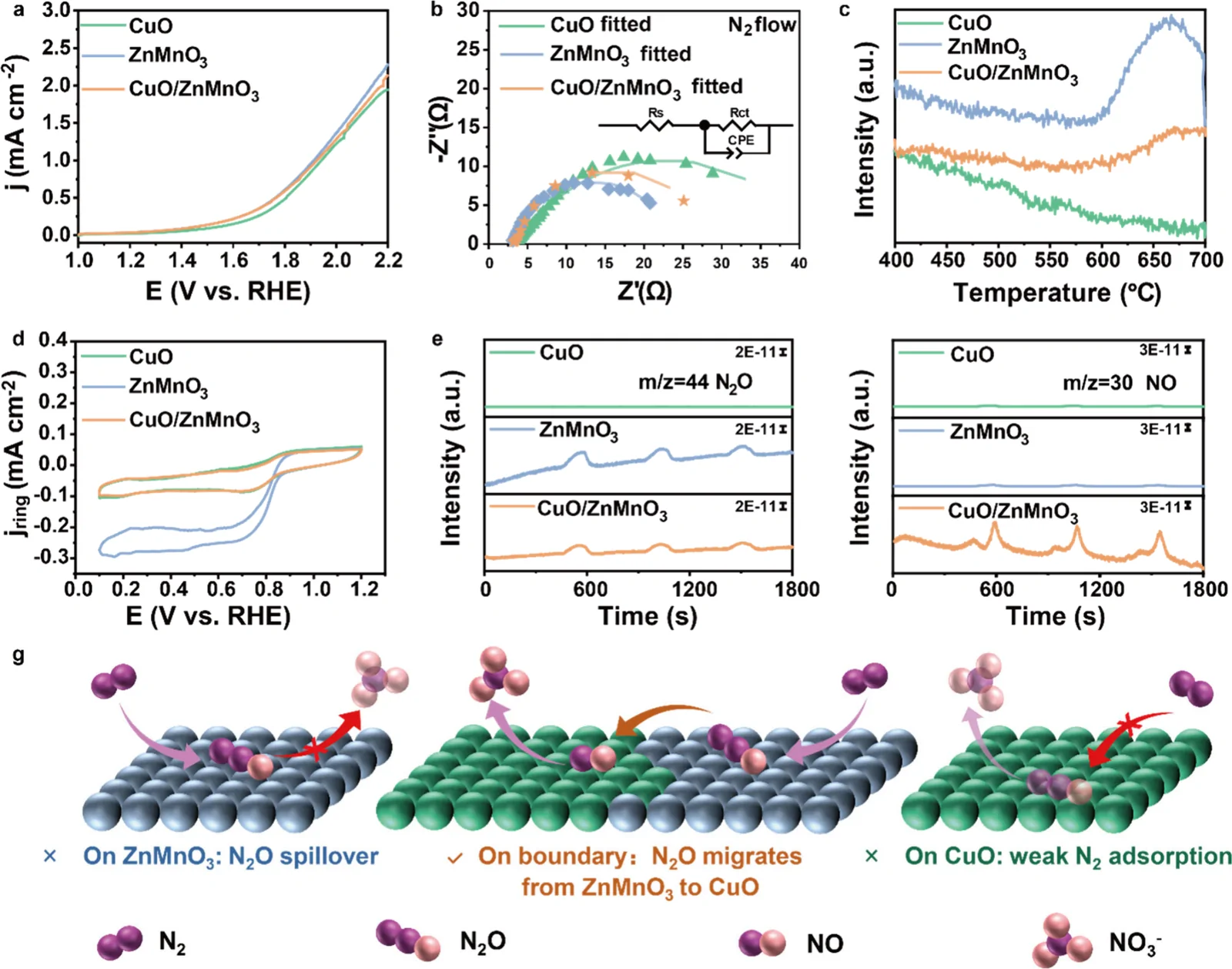
Mechanistic investigation of CuO/ZnMnO_3_ heterostructure catalyst. (a, b, c) LSV curves (a), EIS curves (b), and TPD curves (c) of CuO, ZnMnO_3_, and CuO/ZnMnO_3_ under N_2_ flow. (d) CV curves on Pt ring; GC covered with CuO, ZnMnO_3_, and CuO/ZnMnO_3_ under N_2_ flow. The disc electrode was fixed at 2.1 V vs RHE. RRDE rotation speed is 400 rpm. (e, f) Online DEMS measurements of eNOR at *m*/*z* = 44 (e) and *m*/*z* = 30 (f) over CuO, ZnMnO_3,_ and CuO/ZnMnO_3_. (g) The schematic diagram of the eNOR tandem mechanism over CuO/ZnMnO_3_.

Then, the charge-transfer resistance was measured by electrochemical impedance spectroscopy (EIS) under Ar- and N_2_-saturated conditions. It shows that under Ar atmosphere, CuO had the smallest charge-transfer resistance, aligning with its OER activity. In contrast, ZnMnO_3_ demonstrated a higher resistance (Figure S17). Under N_2_ atmosphere, however, the charge-transfer resistance of CuO increased dramatically to 38.4 Ω, indicating its passivation toward nitrogen-involved reactions. On the other hand, ZnMnO_3_ achieved the lowest resistance (20.4 Ω), while CuO/ZnMnO_3_ registered a resistance of 25.3 Ω. This supports that ZnMnO_3_ serves as the primary site for N_2_ activation (Figure 3b). To obtain direct physical evidence of the N_2_ adsorption and activation capabilities of each component, temperature-programmed desorption of nitrogen (N_2_-TPD) was performed (Figure 3c). Pure CuO exhibits a negligible desorption peak, indicating its chemical inertness toward N_2_. In contrast, both ZnMnO_3_ and the CuO/ZnMnO_3_ heterostructure display pronounced N_2_ desorption profiles at elevated temperatures (spanning 600 to 700 °C). This high desorption temperature signifies a strong chemisorption of N_2_, wherein the vacant orbital configuration of Zn^2+^/Mn^4^^+^ active sites successfully induces electron transfer from N_2_, thereby weakening the N≡N triple bond. These TPD results support ZnMnO_3_ as the primary site for N_2_ chemisorption and initial activation. This gas-phase adsorption capability is further corroborated by in situ electrochemical impedance and capacitance analyses under working conditions. As shown in Figures S18–S20, while the physical double-layer capacitance (C_dl_) of all catalysts remains virtually identical (approximately 0.50 mF cm^–2^) under an inert Ar atmosphere, a stark discrepancy emerges under N_2_-purged electrolysis. Under N_2_, the apparent capacitance of ZnMnO_3_ increases dramatically to 3.39 mF cm^–2^, which is attributed to not only the chemisorption-induced pseudocapacitance arising from the strong orbital coupling and charge transfer between the activated nitrogen species and the Zn/Mn active sites, but the great N_2_ adsorption ability of ZnMnO_3_. Concurrently, the capacitance of CuO remains essentially unchanged (0.56 mF cm^–2^), further validating its inertness to N_2_ under aqueous conditions. Intriguingly, the CuO/ZnMnO_3_ heterostructure exhibits an intermediate capacitance increase, suggesting that the incorporation of CuO does not intrinsically enhance the initial N_2_ adsorption density of the ZnMnO_3_ component. Instead, the substantially boosted overall eNOR efficiency of the heterostructure should originate from downstream kinetic facilitation, which is the efficient tandem capture and subsequent oxidation of migrated intermediates on the neighboring CuO phase.

To elucidate the possible intermediates, rotating-ring-disk-electrode (RRDE) experiments were conducted. Soluble nitrogen oxides were detected on ZnMnO_3_ (reduction current at 0.86 V), whereas they were absent for CuO and CuO/ZnMnO_3_, indicating rapid consumption of intermediates in CuO/ZnMnO_3_ (Figures 3d and S21). In situ DEMS was utilized to trace the evolution of volatile intermediates. As shown in Figure 3e, distinct generation of N_2_O (*m*/*z* = 44) was detected on both ZnMnO_3_ and CuO/ZnMnO_3_ as the applied potential increased under a N_2_ flow, whereas the N_2_O signal for CuO was negligible. More importantly, pronounced NO (*m*/*z* = 30) evolution was observed exclusively for the CuO/ZnMnO_3_ heterostructure (Figure 3f). These evolution trends support that N_2_O and NO are key intermediates, underscoring the critical role of the CuO component in further oxidizing the N_2_O intermediate supplied by ZnMnO_3_ (Figure S22).

Collectively, these results support a stepwise tandem mechanism for CuO/ZnMnO_3_ (Figure 3g). First, N_2_ adsorption and primary oxidation predominantly occur on ZnMnO_3_, where Zn/Mn sites and oxygen vacancy-associated electronic structures provide favorable environments for N_2_ activation. The initially formed N_2_O intermediates then further oxidized to NO and ultimately nitrate. The initially formed N_2_O intermediate then migrates toward the adjacent CuO domain across the heterointerface, where it is further oxidized to NO and ultimately to nitrate. The spatial decoupling of N_2_ activation and downstream intermediate oxidation circumvents the limitations associated with single-site scaling relations, thereby enabling an efficient eNOR. Although the combined kinetic analysis, RRDE, in situ DEMS, in situ Raman, DFT calculations, and AIMD simulations provide self-consistent support for the proposed tandem pathway, alternative or parallel oxidation routes cannot be completely excluded in such a complex multielectron system.

### DFT Calculations

To further examine the phase evolution and interface stability of CuO/ZnMnO_3_ under anodic eNOR conditions, in situ Raman spectroscopy was conducted at 2.1 V vs RHE in N_2_-saturated 1 M KOH. At the initial stage, Raman bands at 296, 340, 601, and 645 cm^–1^ were observed (Figure S23). The bands at 296 and 340 cm^–1^ can be associated with lattice vibrations of the parent CuO/ZnMnO_3_ oxide framework, with CuO-related Raman-active modes typically appearing in the low-frequency region around 290–340 cm^–1^.^40^ The band at 645 cm^–1^ is related to Mn–O stretching vibrations in oxidized MnO_6_ octahedral units, which are commonly observed in the 630–665 cm^–1^ region for manganese oxides.^41^ The band at 601 cm^–1^ is likely associated with an initial oxygen-deficient M–O coordination environment in the parent CuO/ZnMnO_3_ oxide surface.^40,42^ During anodic polarization, the 296 and 340 cm^–1^ bands gradually weakened, and the 601 cm^–1^ band disappeared after around 150 s, indicating substantial perturbation of the original surface metal–oxygen coordination. In contrast, the 645 cm^–1^ band continuously intensified, accompanied by the emergence of a sharp band at around 527 cm^–1^ and a shoulder at ca. 480 cm^–1^ (Figure S24). Because Raman features of MnO_6_-based manganese oxides and layered phyllomangenates mainly appear in the 400–650 cm^–1^ region, the enhanced 645 cm^–1^ band can be assigned to Mn–O stretching vibrations in oxidized MnO_6_ octahedral units, while the newly formed 527 cm^–1^ band and the 480 cm^–1^ shoulder are tentatively attributed to Mn–O–Mn/Mn–OH vibrations in reconstructed Mn-rich oxyhydroxide/birnessite-like MnO_*x*_ surface species.^41,43^ These spectral changes indicate that CuO/ZnMnO_3_ undergoes partial surface reconstruction under eNOR operating conditions, generating an active oxide-derived Mn-rich surface layer while retaining the underlying oxide heterostructure as the structural and electronic scaffold. Moreover, the HRTEM image of the catalyst after electrolysis supports that the CuO/ZnMnO_3_ heterointerface is largely preserved after long-term operation, while only the bulk ZnMnO_3_ phase undergoes amorphization (Figure S25). This suggests that the catalyst maintains a stable heterointerface scaffold while forming a partial oxide-derived reconstructed surface layer during eNOR. Therefore, the working catalyst can be described as a reconstructed surface layer supported by a largely preserved crystalline CuO/ZnMnO_3_ heterostructure scaffold.

To gain atomic-level insights into the intrinsic roles of oxygen vacancies and the CuO/ZnMnO_3_ heterointerface, DFT calculations were performed on oxygen-deficient ZnMnO_3_ and CuO oxide surfaces. We note that the in situ Raman results reveal partial reconstruction of the outer surface under anodic eNOR conditions, forming oxide-derived Mn-rich MnO_*x*_/MnOOH-like species. Therefore, the present DFT models are not intended to serve as complete atomic representations of the fully reconstructed operando surfaces. Rather, they are used to clarify the fundamental functions of the parent oxygen-deficient oxide frameworks. On the basis of our electrochemical measurements, which strongly suggest a stepwise reaction pathway,^22^ we modeled the elementary steps involved in the conversion of N_2_ to nitrate (Figure S26). The calculations support the feasibility of the proposed tandem pathway involving (i) initial N_2_ adsorption; (ii) activation of N_2_ on oxygen-deficient ZnMnO_3_ phase to N_2_O; (iii) subsequent N_2_O migration from ZnMnO_3_ phase to CuO phase; (iv) N_2_O oxidation to NO on oxygen-deficient CuO phase; and (v) NO oxidation to nitrate on oxygen-deficient CuO phase.

The computed energy profiles (Figures 4a and S27) reveal that the oxidation of N_2_O to HONNO (a key intermediate preceding N–N bond cleavage) is supposed to be the potential-determining step (PDS) for the entire 10-electron transfer process. Notably, the calculated reaction energy barrier for this critical step is drastically different across the two components. It is as high as 3.00 eV on the ZnMnO_3_ (220) surface, but is significantly lower at 2.66 eV on the CuO (002) surface. This substantial 0.34 eV reduction in the energy barrier underscores the pivotal role of the heterostructure in facilitating this energetically demanding step. The lower barrier on CuO is attributed to its more favorable electronic structure, which stabilizes the transition state of N_2_O oxidation by enabling efficient electron transfer and optimal alignment of the O 2p and N 2p orbitals during O insertion.^44^ Furthermore, our calculations explain the experimental observation of NO as a detectable intermediate. We identified that the subsequent oxidation of the NO formed on the CuO surface to HNO_2_ is subject to a substantial energy barrier of 2.57 eV. This relatively high barrier leads to a kinetic bottleneck, resulting in the temporary accumulation of NO species before their further oxidation—a finding that correlates exceptionally well with the NO signal detected in our in situ DEMS experiments.

**Figure 4:**
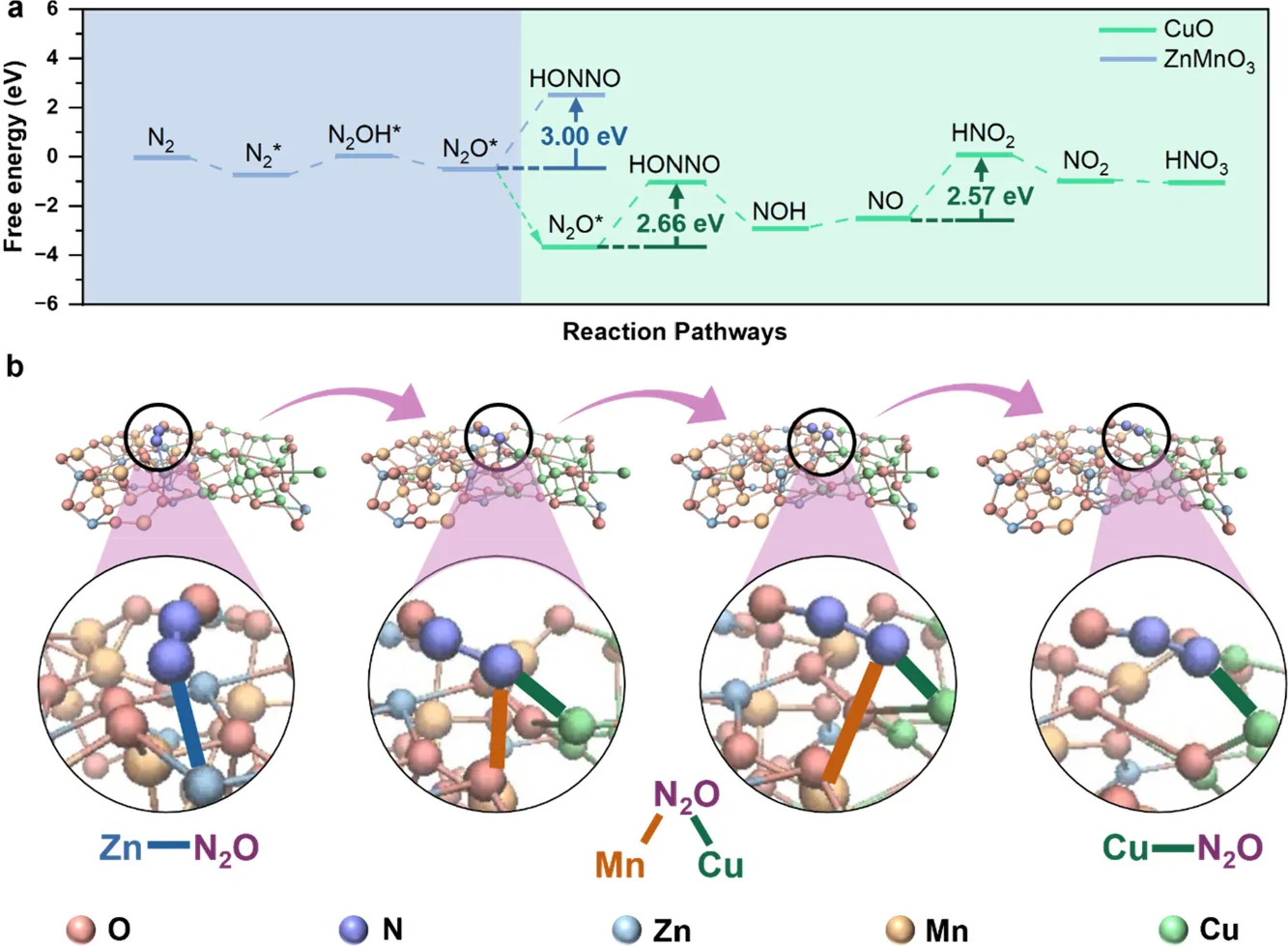
DFT and AIMD calculations of CuO/ZnMnO_3_ heterostructure catalyst during eNOR process. (a) Free energy diagram of eNOR over ZnMnO_3_ (the initial 2e^–^ conversion) and CuO (the subsequent 8e^–^ conversion). (b) Schematic diagram of N_2_O thermodynamic migration from ZnMnO_3_ to CuO from AIMD simulations.

To provide a comprehensive mechanistic insight into the entire reaction pathway, the thermodynamic feasibility of N_2_O migration between the two phases was assessed using Ab initio molecular dynamics (AIMD) simulations (Figure 4b). The AIMD trajectories provide a dynamic indication that N_2_O can approach and interact with the CuO domain across the ZnMnO_3_–CuO boundary within the simulation window, suggesting that interfacial transfer of N_2_O is dynamically feasible in the modeled heterostructure at 298 K. The preferred pathway involves N_2_O transferring from ZnMnO_3_ to CuO through interactions at the interface, facilitated by surface oxygen vacancies on CuO. This spontaneous migration of the key intermediate (N_2_O) from the activation site (ZnMnO_3_) to the oxidation site (CuO) effectively decouples the two catalytic functions, preventing the accumulation of energy-intensive steps on a single material and thereby significantly lowering the overall reaction barrier. While the combined DFT energetics, AIMD trajectories and in situ DEMS/RRDE observations (notably the detection of N_2_O and NO) provide strong and self-consistent support for the ZnMnO_3_–(N_2_O)–CuO tandem pathway as the dominant route in our system, we acknowledge that alternative or parallel pathways cannot be completely excluded.

The DFT and AIMD calculations provide theoretical support for the proposed tandem mechanisms. They quantitatively explain the superior activity of the CuO/ZnMnO_3_ heterostructure by highlighting the dramatic reduction of the rate-determining energy barrier on CuO and rationalizing the observed reaction intermediates. The spontaneous interfacial diffusion of N_2_O further underscores the design principle of using heterostructures with complementary functions for efficient multistep electrocatalytic reactions, such as eNOR. Together with the in situ Raman results, these calculations provide a complementary description of the working catalyst. The Raman spectra reveal the dynamic reconstruction of the outer surface into oxide-derived Mn-rich MnO_*x*_/MnOOH-like species under anodic eNOR conditions, whereas the theoretical calculations clarify the intrinsic roles of oxygen vacancies and heterointerfacial coupling in the parent CuO/ZnMnO_3_ oxide framework. Therefore, the reconstructed surface layer and the underlying oxygen-deficient oxide heterostructure could be considered an integrated working system.

## Conclusion

In summary, we report a CuO/ZnMnO_3_ heterostructure catalyst, synthesized via a citrate-assisted sol–gel method, as an efficient catalyst for direct eNOR in alkaline media. This catalyst achieves an impressive nitrate yield rate of 52.1 μmol h^–1^ mg^–1^ with an FE of 45.2%, outperforming single-component benchmarks while maintaining excellent stability over 48 h. Energy profiles and AIMD simulations reveal a tandem pathway in which oxygen-deficient ZnMnO_3_ initiates the activation of N_2_ to N_2_O, and oxygen-deficient CuO facilitates its subsequent oxidation. This pathway enables spontaneous migration of N_2_O across the heterointerface and reduces the energy barrier by 0.34 eV by decoupling the two critical catalytic functions, thereby accelerating the overall process. This work not only demonstrates the high activity of CuO/ZnMnO_3_ catalyst for eNOR but also offers a design strategy for engineering efficient heterogeneous catalysts via the integration of tandem sites.

## Supplementary Material



## Notes

Additional SEM, TEM, XRD, XPS, electrochemistry data, and computational details (PDF)

## References

[1] P F. M. (1911). Fixation
of Atmospheric Nitrogen. Nature.

[2] Marx J. L. (1974). Nitrogen
Fixation: Research Efforts Intensify. Science.

[3] Canfield D. E., Glazer A. N., Falkowski P. G. (2010). The Evolution and Future of Earth’s
Nitrogen Cycle. Science.

[4] Fulweiler R. W., Rinehart S., Taylor J., Kelly M. C., Berberich M. E., Ray N. E., Oczkowski A., Balint S., Benavides M., Church M. J. (2025). Global
importance of nitrogen fixation across
inland and coastal waters. Science.

[5] Kyriakou V., Garagounis I., Vourros A., Vasileiou E., Stoukides M. (2020). An Electrochemical Haber-Bosch Process. Joule.

[6] Liu X., Elgowainy A., Wang M. (2020). Life cycle energy use and greenhouse
gas emissions of ammonia production from renewable resources and industrial
by-products. Green Chem..

[7] Li D., Zan L., Chen S., Shi Z., Chen P., Xi Z., Deng D. (2022). Direct conversion of
N_2_ and O_2_: status, challenge
and perspective. National Science Review.

[8] Wang Y., Yu Y., Jia R., Zhang C., Zhang B. (2019). Electrochemical synthesis
of nitric acid from air and ammonia through waste utilization. National Science Review.

[9] Guo Y., Zhang S., Zhang R., Wang D., Zhu D., Wang X., Xiao D., Li N., Zhao Y., Huang Z. (2022). Electrochemical Nitrate
Production via Nitrogen Oxidation
with Atomically Dispersed Fe on N-Doped Carbon Nanosheets. ACS Nano.

[10] Yan Q., Zhao R., Yu L., Zhao Z., Liu L., Xi J. (2024). Enhancing Compatibility of Two-Step Tandem Catalytic Nitrate Reduction
to Ammonia Over P-Cu/Co­(OH)_2_. Adv.
Mater..

[11] Yuan Y., Qian X., Liu S., Chen W., Lu M., Delmo E. P., Zhang Y., Yao Y., Lyu Y., Liu Y. (2026). Spatially decoupled La ZnO core shell structure tunes
N_2_ activation and suppresses oxygen evolution. Chem. Catalysis.

[12] Dai C., Sun Y., Chen G., Fisher A. C., Xu Z. J. (2020). Electrochemical
Oxidation of Nitrogen towards Direct Nitrate Production on Spinel
Oxides. Angew. Chem., Int. Ed..

[13] Zhang S., Rong X., Sun T., Gao P., Liu J., Qiu X., Zhou X., Wu Z. (2022). Enhancement of N2 adsorption by Z-scheme
porous g-C_3_N_4_/ZnFe_2_O_4_ composite
material for high-efficient photocatalytic nitrogen fixation. J. Porous Mater..

[14] Jang D. M., Kwak I. H., Kwon E. L., Jung C. S., Im H. S., Park K., Park J. (2015). Transition-Metal
Doping of Oxide
Nanocrystals for Enhanced Catalytic Oxygen Evolution. J. Phys. Chem. C.

[15] Ge X., Yuan J., Li H., Wang L., Xie H., Liu C. (2025). Enhancing *CO and *N≡N
Orbital Coupling via Tuned Mn d-p Hybridization
at MnO/Mn_7_C_3_ Interfaces for Highly Selective
Urea Electrosynthesis. Adv. Energy Mater..

[16] Li T., Han S., Wang C., Huang Y., Wang Y., Yu Y., Zhang B. (2021). Ru-Doped Pd Nanoparticles for Nitrogen Electrooxidation to Nitrate. ACS Catal..

[17] Xu F., Liu X., Zhang L., Guo M., Li M., Ding X., Zhang L. (2023). Revealing and optimizing
the dialectical relationship between NOR
and OER: cation vacancy engineering enables RuO_2_ with unanticipated
high electrochemical nitrogen oxidation performance. Adv. Energy Mater..

[18] Zheng H., Ma Z., Liu Y., Zhang Y., Ye J., Debroye E., Zhang L., Liu T., Xie Y. (2024). Perovskite oxide as
a new platform for efficient electrocatalytic nitrogen oxidation. Angew. Chem., Int. Ed..

[19] Zhang L., Cong M., Ding X., Jin Y., Xu F., Wang Y., Chen L., Zhang L. (2020). A Janus Fe-SnO_2_ Catalyst that Enables Bifunctional Electrochemical Nitrogen
Fixation. Angew. Chem., Int. Ed..

[20] Zeng H., Wang D., Yang C.-J., Dong C.-L., Lin W., Sang X., Yang B., Li Z., Yao S., Zhang Q. (2025). Interfacial Electron Engineering Unlocks Efficient
Nitrate Electrosynthesis by Balancing Nitrogen Activation and Oxygen
Evolution. ACS Catal..

[21] Han S., Wang C., Wang Y., Yu Y., Zhang B. (2021). Electrosynthesis
of Nitrate via the Oxidation of Nitrogen on Tensile-Strained Palladium
Porous Nanosheets. Angew. Chem., Int. Ed..

[22] Wang Y., Li T., Yu Y., Zhang B. (2022). Electrochemical synthesis of nitric
acid from nitrogen oxidation. Angew. Chem.,
Int. Ed..

[23] Kuang M., Wang Y., Fang W., Tan H., Chen M., Yao J., Liu C., Xu J., Zhou K., Yan Q. (2020). Efficient
Nitrate Synthesis via Ambient Nitrogen Oxidation with Ru-Doped TiO_2_/RuO_2_ Electrocatalysts. Adv.
Mater..

[24] Li Q., Xiao Z., Jia W., Li Q., Li X., Wang W. (2023). Copper nanowires decorated
with TiO_2‑x_ from MXene
for enhanced electrocatalytic nitrogen oxidation into nitrate under
vacuum assistance. Nano Research.

[25] Zheng X., Wu H., Gao Y., Chen S., Xue Y., Li Y. (2024). Controllable
assembly of highly oxidized cobalt on graphdiyne surface for efficient
conversion of nitrogen into nitric acid. Angew.
Chem., Int. Ed..

[26] Nie Z., Zhang L., Ding X., Cong M., Xu F., Ma L., Guo M., Li M., Zhang L. (2022). Catalytic Kinetics
Regulation for Enhanced Electrochemical Nitrogen Oxidation by Ru-Nanoclusters-Coupled
Mn_3_O_4_ Catalysts Decorated with Atomically Dispersed
Ru Atoms. Adv. Mater..

[27] Fang W., Du C., Kuang M., Chen M., Huang W., Ren H., Xu J., Feldhoff A., Yan Q. (2020). Boosting efficient ambient nitrogen
oxidation by a well-dispersed Pd on MXene electrocatalyst. Chem. Commun..

[28] Kresse G., Furthmüller J. (1996). Efficient
iterative schemes for ab initio total-energy
calculations using a plane-wave basis set. Phys.
Rev. B.

[29] Kresse G., Furthmüller J. (1996). Efficiency of ab-initio total energy
calculations for
metals and semiconductors using a plane-wave basis set. Comput. Mater. Sci..

[30] Blöchl P. E. (1994). Projector
augmented-wave method. Phys. Rev. B.

[31] Grimme, S. ; Antony, J. ; Ehrlich, S. ; Krieg, H. A consistent and accurate ab initio parametrization of density functional dispersion correction (DFT-D) for the 94 elements H-Pu. J. Chem. Phys. 2010, 132 (15). 10.1063/1.3382344. 20423165

[32] Humphrey W., Dalke A., Schulten K. (1996). VMD: Visual molecular dynamics. J. Mol. Graphics.

[33] Wang Q., Li J., Wang Q., Tan H., Tian Y., Wang C., Si W., Xu Y., Yan X., Liang J. (2025). Interface Engineering
of NiO/CuO Heterojunction Enabling Built-In Electric Field for Efficient
Electrocatalytic Ammonia Oxidation Reaction. ACS Appl. Mater. Interfaces.

[34] Rettenmaier K., Zickler G. A., Redhammer G. J., Berger T. (2023). Substrate-Enabled Room-Temperature
Electrochemical Deposition of Crystalline ZnMnO3. ChemPhysChem.

[35] Fu Y.-C., Chen Y.-C., Wu C.-M., Hsiao V. K. S. (2023). Tailored Nanoscale
Architectures for White Light Photoelectrochemistry: Zinc Oxide Nanorod-Based
Copper Oxide Heterostructures. Coatings.

[36] Chastain J., King R. C. (1992). Handbook of X-ray photoelectron spectroscopy. Perkin-Elmer Corporation.

[37] Muilenberg, G. Handbook of X-ray Photoelectron Spectroscopy; Perkin-Elmer Corp.: Eden Prarie, MN, 1979.

[38] Du Z., Yang K., Du H., Li B., Wang K., He S., Wang T., Ai W. (2023). Facile and Scalable Synthesis of
Self-Supported Zn-Doped CuO Nanosheet Arrays for Efficient Nitrate
Reduction to Ammonium. ACS Appl. Mater. Interfaces.

[39] Zhang Y., Du F., Wang R., Ling X., Wang X., Shen Q., Xiong Y., Li T., Zhou Y., Zou Z. (2021). Electrocatalytic
fixation of N_2_ into NO_3_
^–^:
electron transfer between oxygen vacancies and loaded Au in Nb_2_O_5‑x_ nanobelts to promote ambient nitrogen
oxidation. Journal of Materials Chemistry A.

[40] Deng Y., Handoko A. D., Du Y., Xi S., Yeo B. S. (2016). In Situ
Raman Spectroscopy of Copper and Copper Oxide Surfaces during Electrochemical
Oxygen Evolution Reaction: Identification of CuIII Oxides as Catalytically
Active Species. ACS Catal..

[41] Post J. E., McKeown D. A., Heaney P. J. (2021). Raman spectroscopy
study of manganese
oxides: Layer structures. Am. Mineral..

[42] Yin B., Zhang S., Jiang H., Qu F., Wu X. (2015). Phase-controlled
synthesis of polymorphic MnO2 structures for electrochemical energy
storage. Journal of Materials Chemistry A.

[43] Xiao W., Wang D., Lou X. W. (2010). Shape-Controlled
Synthesis of MnO_2_ Nanostructures with Enhanced Electrocatalytic
Activity for
Oxygen Reduction. J. Phys. Chem. C.

[44] Suo W., Sun S., Liu N., Li X., Wang Y. (2020). The adsorption and
dissociation of N_2_O on CuO(111) surface: The effect of
surface structures. Surf. Sci..

