# Supplementary material for: Tandem Catalysis in CuO/ZnMnO3 Heterostructures for Efficient Electrooxidation of N2 to Nitrate
Source: J Am Chem Soc. 2026 Aug 26;148(35):38205–14. doi: 10.1021/jacs.6c10955 (PMC13564438; doi:10.1021/jacs.6c10955)
Supplement: Supplementary file 1 [file ja6c10955_si_001.pdf]

Supporting Information for

Tandem Catalysis in CuO/$ZnMnO_3$ Heterostructures for Efficient Electrooxidation of N_2 to Nitrate

Yuan Yuan[1, #], *Xitang Qian*[1, #], *Shiyuan Liu*[1], *Qingbo Wa*[2], *Yinuo Wang*[1], *Xiaoyi Qiu*[1], *Yan Zhang*[1], *Siqi Lu*[1], *Yuxiang Lyu*[1], *Hua Zhang*[2], *Minhua Shao*[1, 3, 4,] *

1. Department of Chemical and Biological Engineering, The Hong Kong University of Science and Technology, Clear Water Bay, Kowloon, Hong Kong, China

2. Department of Chemistry, City University of Hong Kong, Hong Kong 999077, China

3. CIAC-HKUST Joint Laboratory for Hydrogen Energy, The Hong Kong University of Science and Technology, Clear Water Bay, Kowloon, Hong Kong, China

4. Fok Ying Tung Research Institute, The Hong Kong University of Science and Technology, Guangzhou 511458, China

# These authors contribute equally to this work.

Corresponding Author: kemshao@ust.hk

Table of contents:

1. Supplementary Figures (Figure S1 to S27)
2. Supplementary Tables (Table S1 to S5)
3. References

1. Supplementary Figures

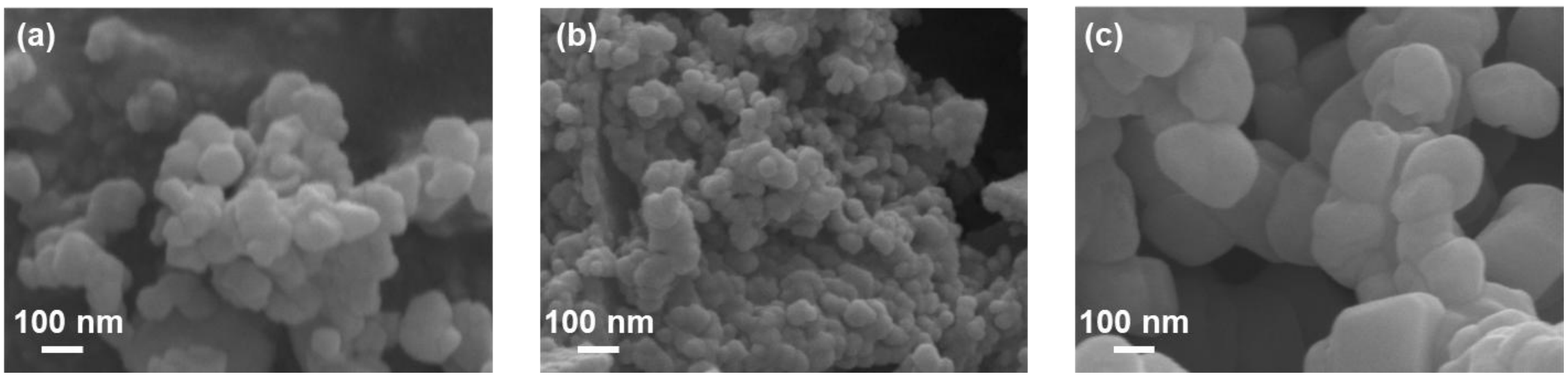

Figure S1. SEM images of **(a)** CuO, **(b)** $ZnMnO_3$, and **(c)** $CuO/ZnMnO_3$.

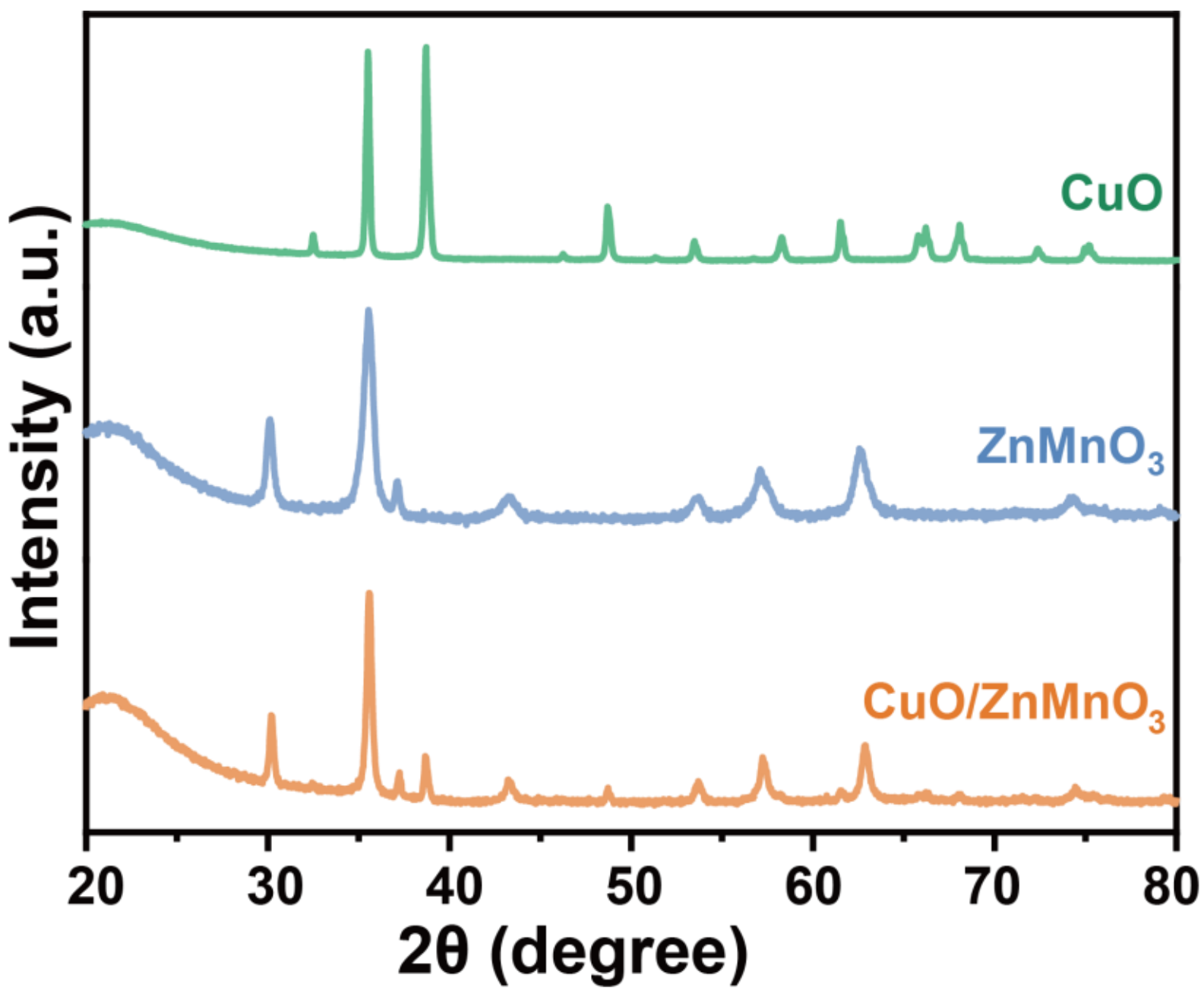

Figure S2. XRD patterns of synthesized CuO, $ZnMnO_3$ and CuO/$ZnMnO_3$.

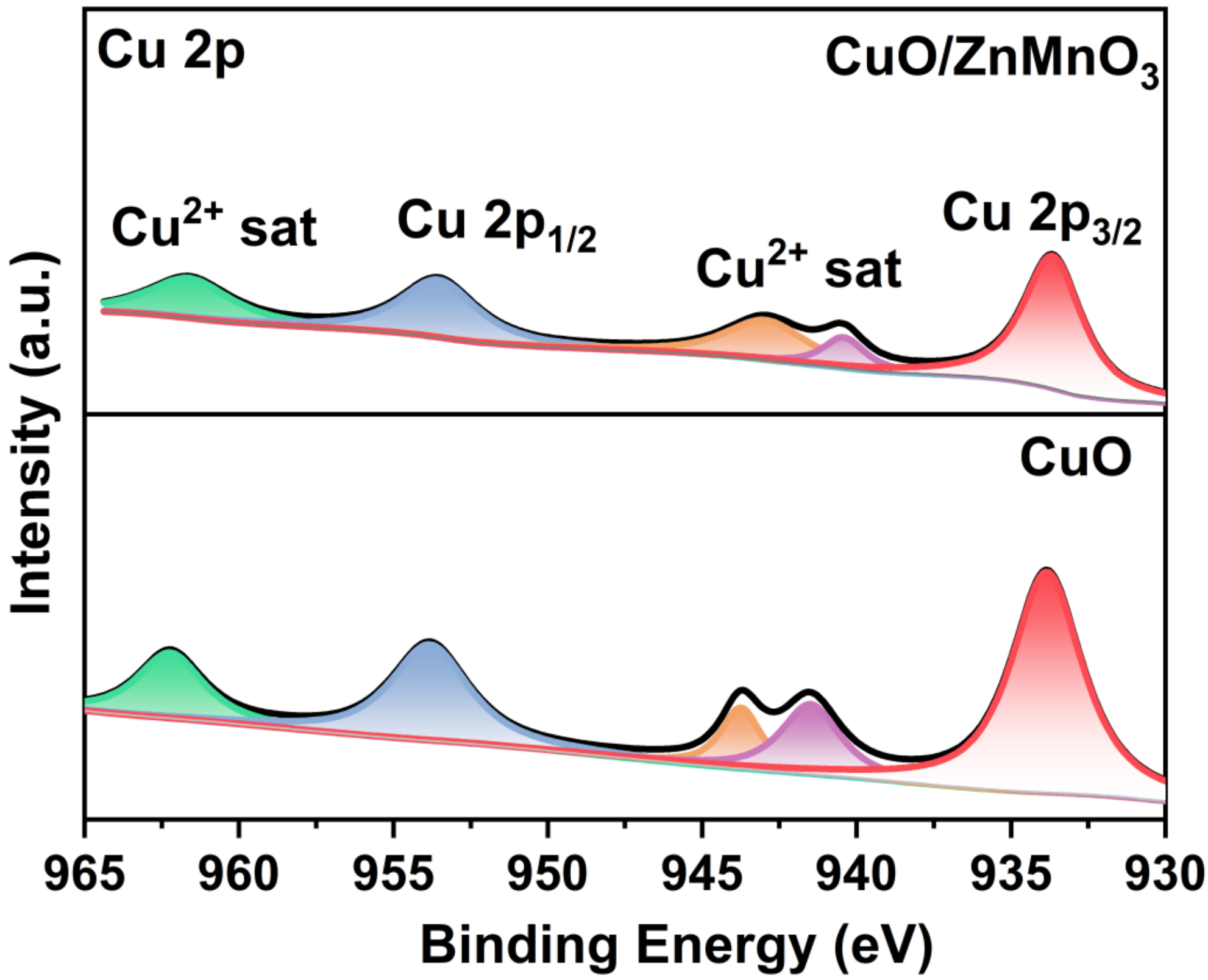

Figure S3. Cu 2p XPS spectrum of CuO and CuO/$ZnMnO_3$.

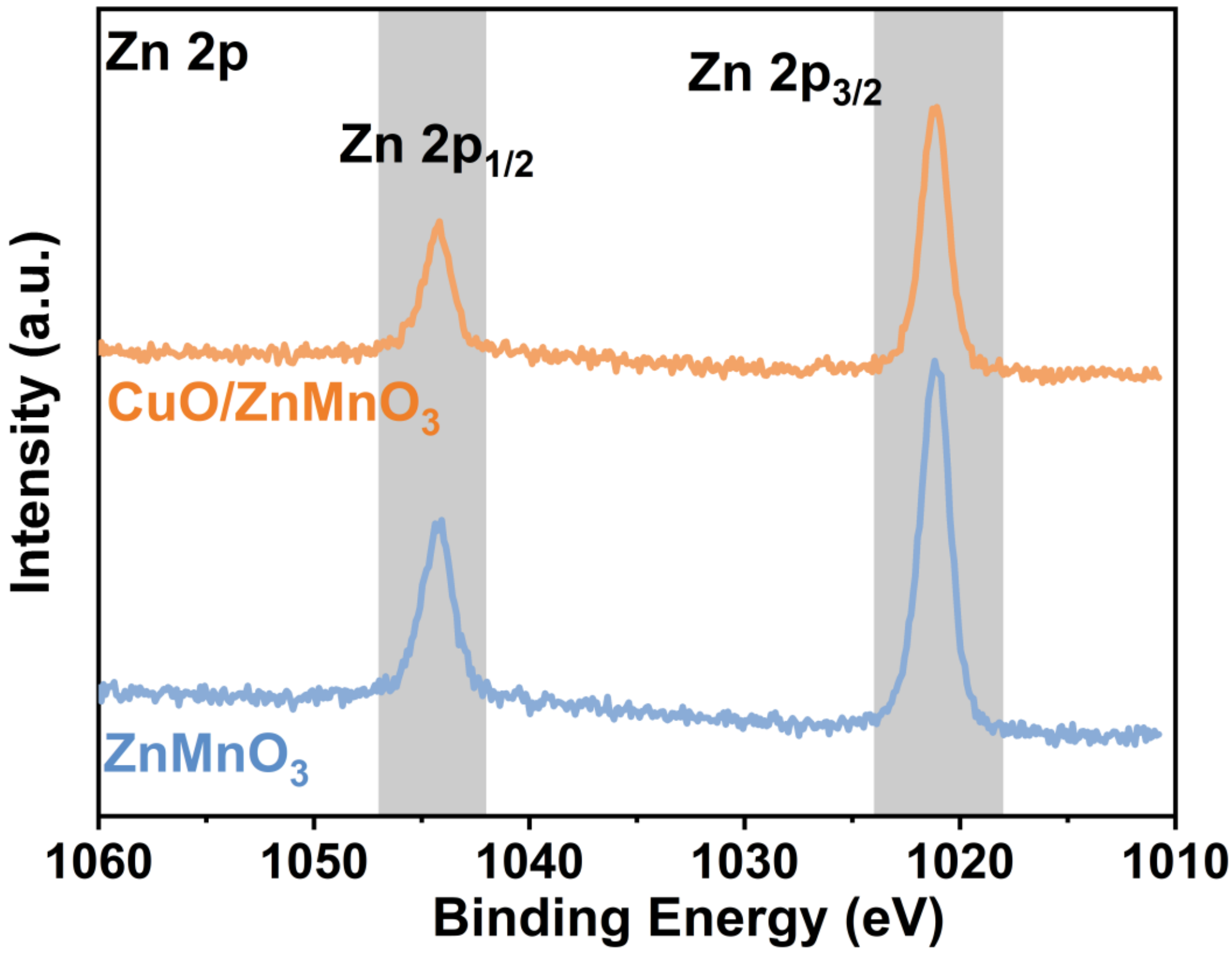

Figure S4. Zn 2p XPS spectrum of $ZnMnO_3$ and $CuO/ZnMnO_3$.

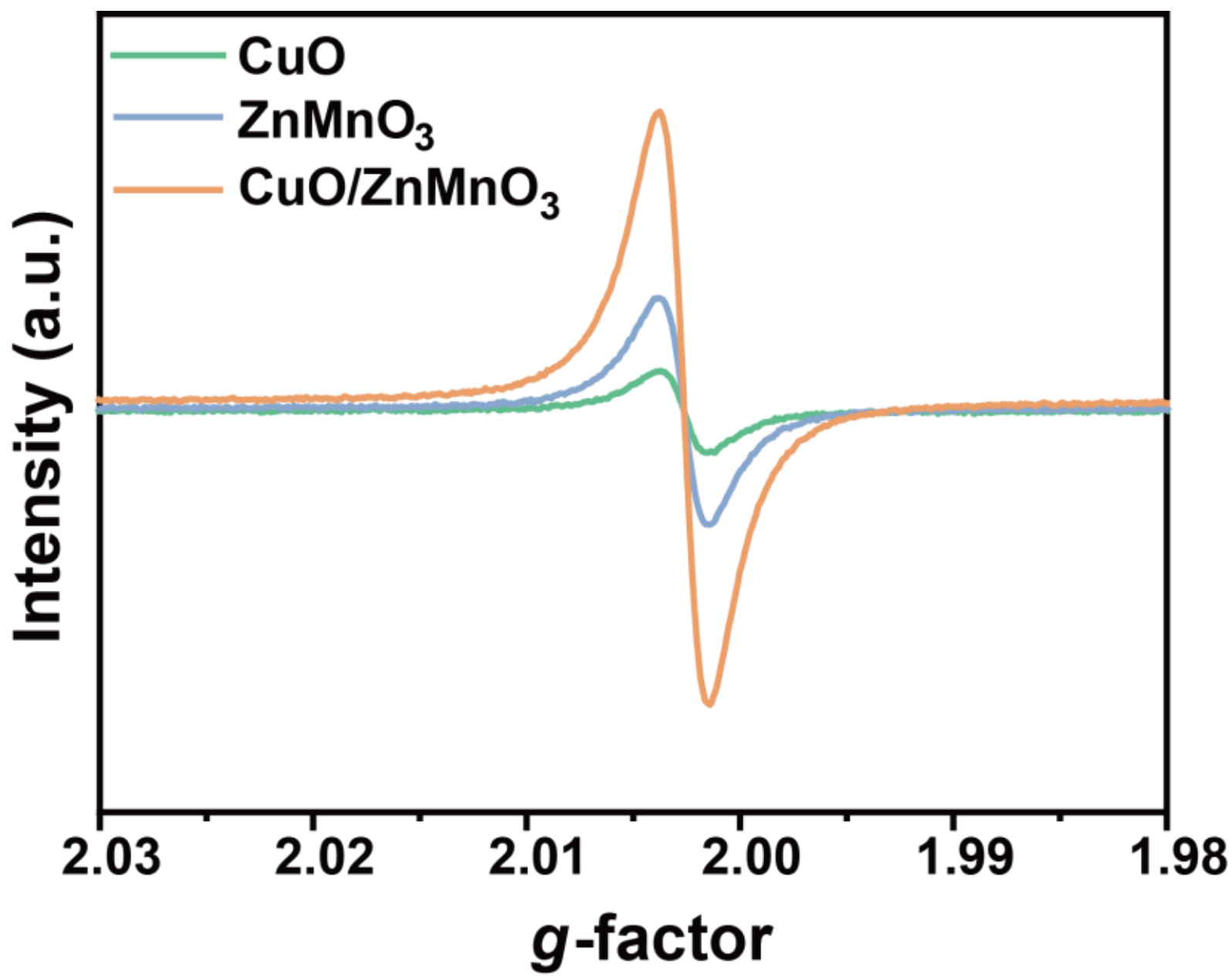

Figure S5. EPR results of CuO, $ZnMnO_3$ and CuO/$ZnMnO_3$.

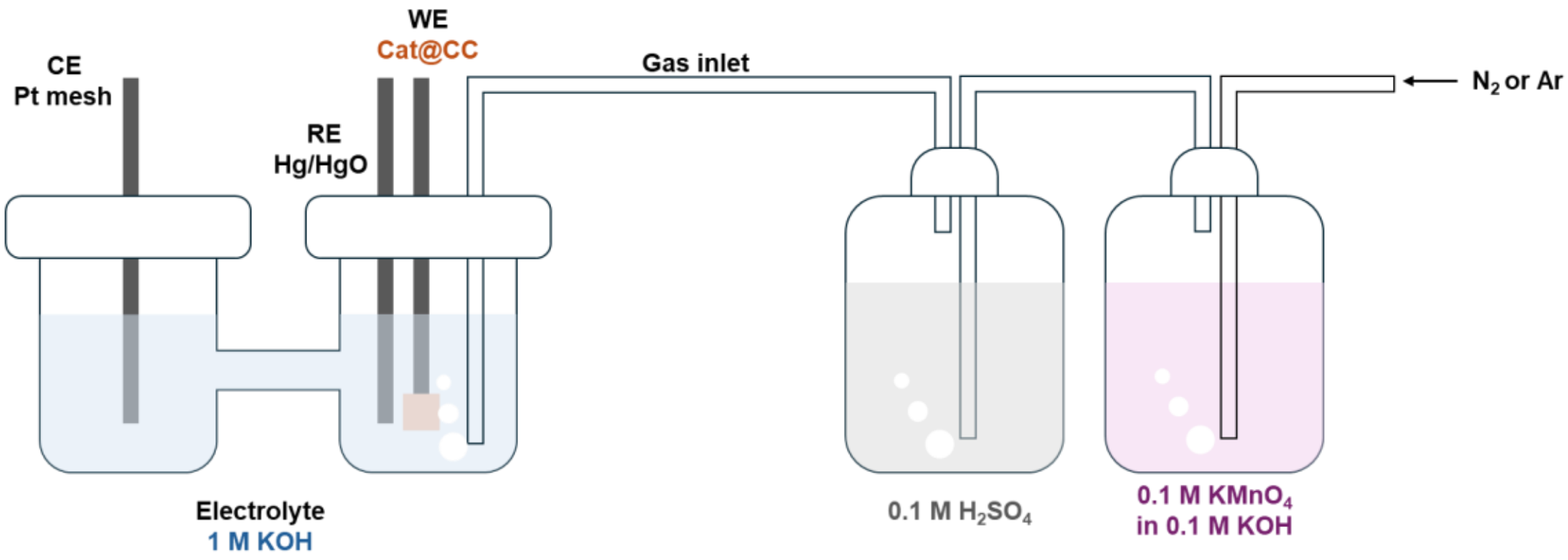

Figure S6. The scheme of an H-type cell for the eNOR experiment.

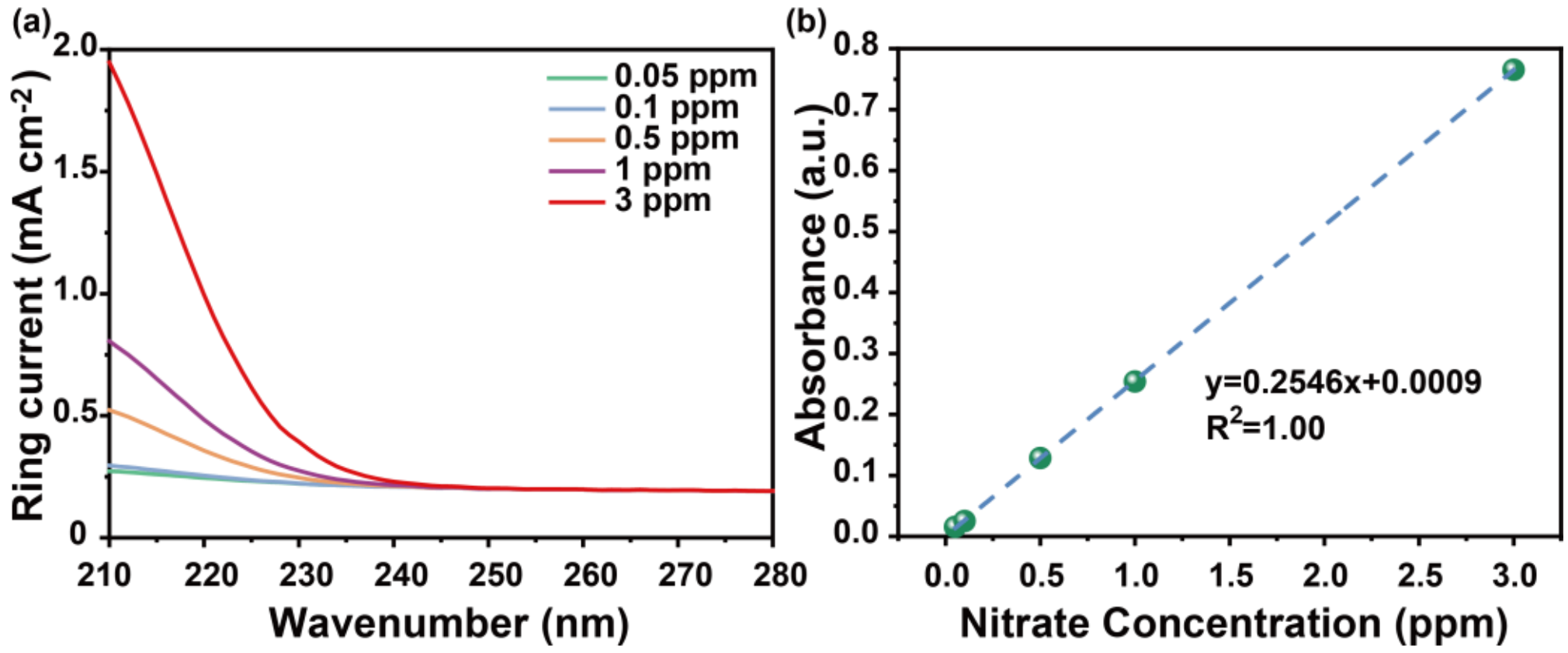

Figure S7. (**a**) UV-vis absorption spectra and (**b**) calibration curves of a standard solution with given concentrations of NO_3^-, respectively.

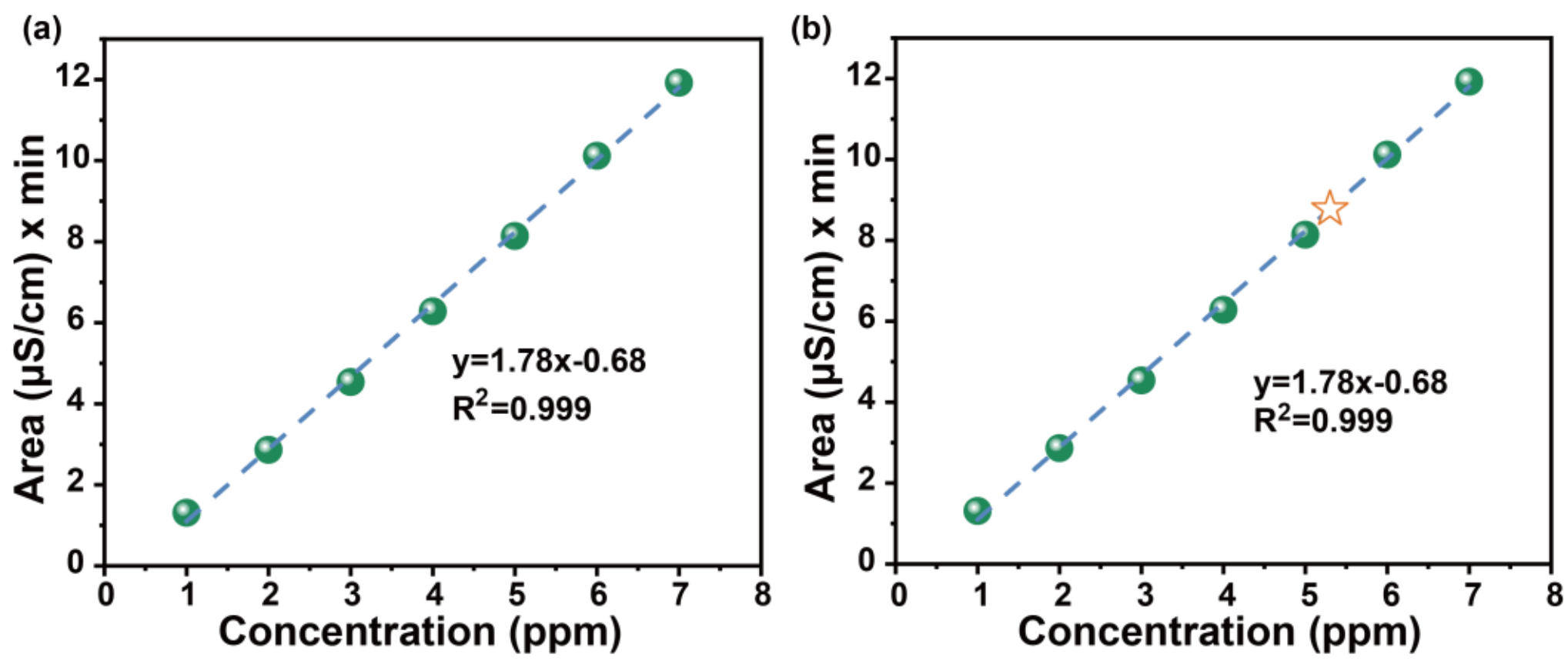

Figure S8. (**a**) IC calibration curve of the standard solution with given nitrate concentrations and (**b**) our work data derived from NOR test over $CuO/ZnMnO_3$ at 2.1 V *vs.* RHE.

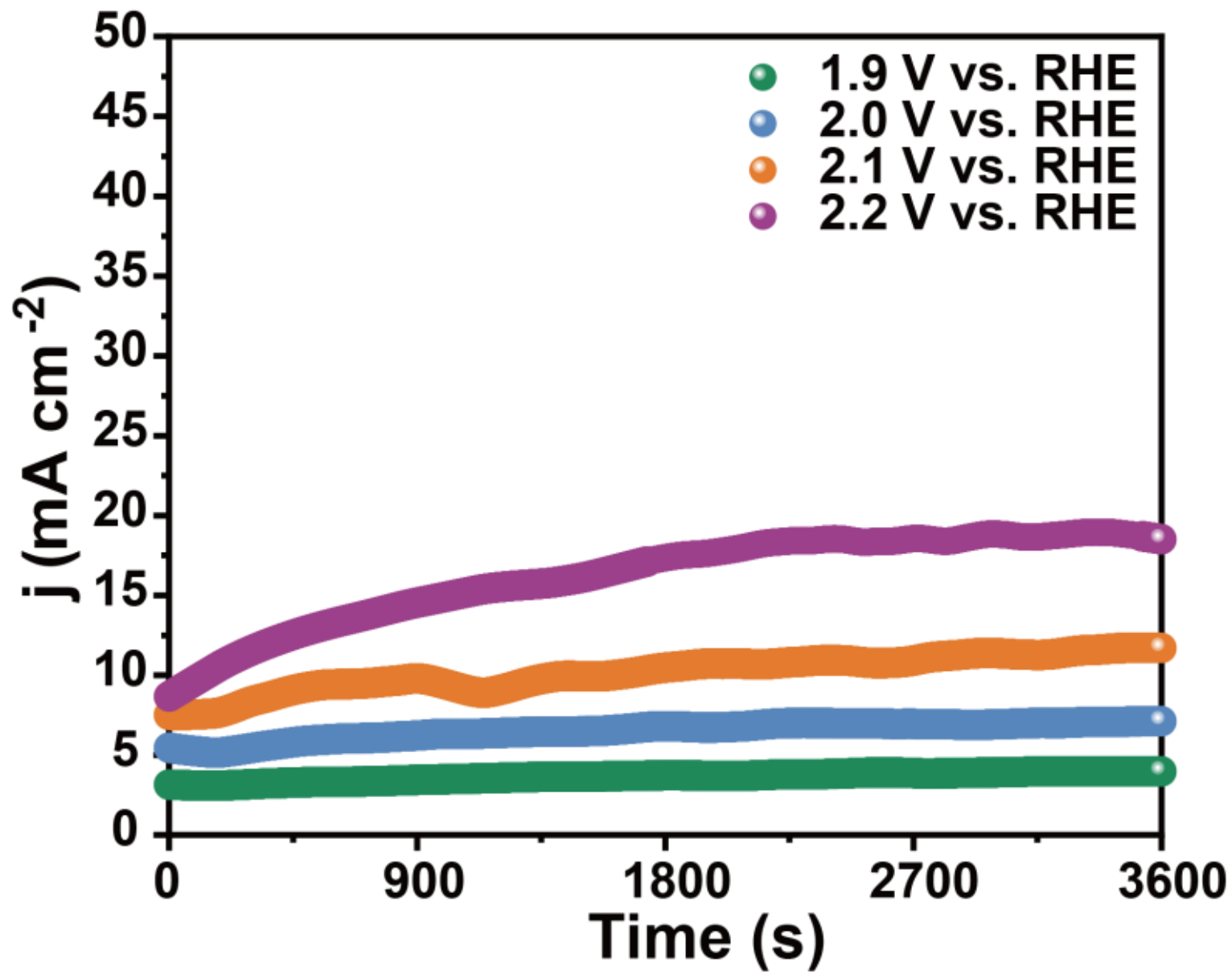

Figure S9. Chronoamperometry curves of $CuO/ZnMnO_3$ obtained in N_2-saturated 1 M KOH aqueous electrolyte.

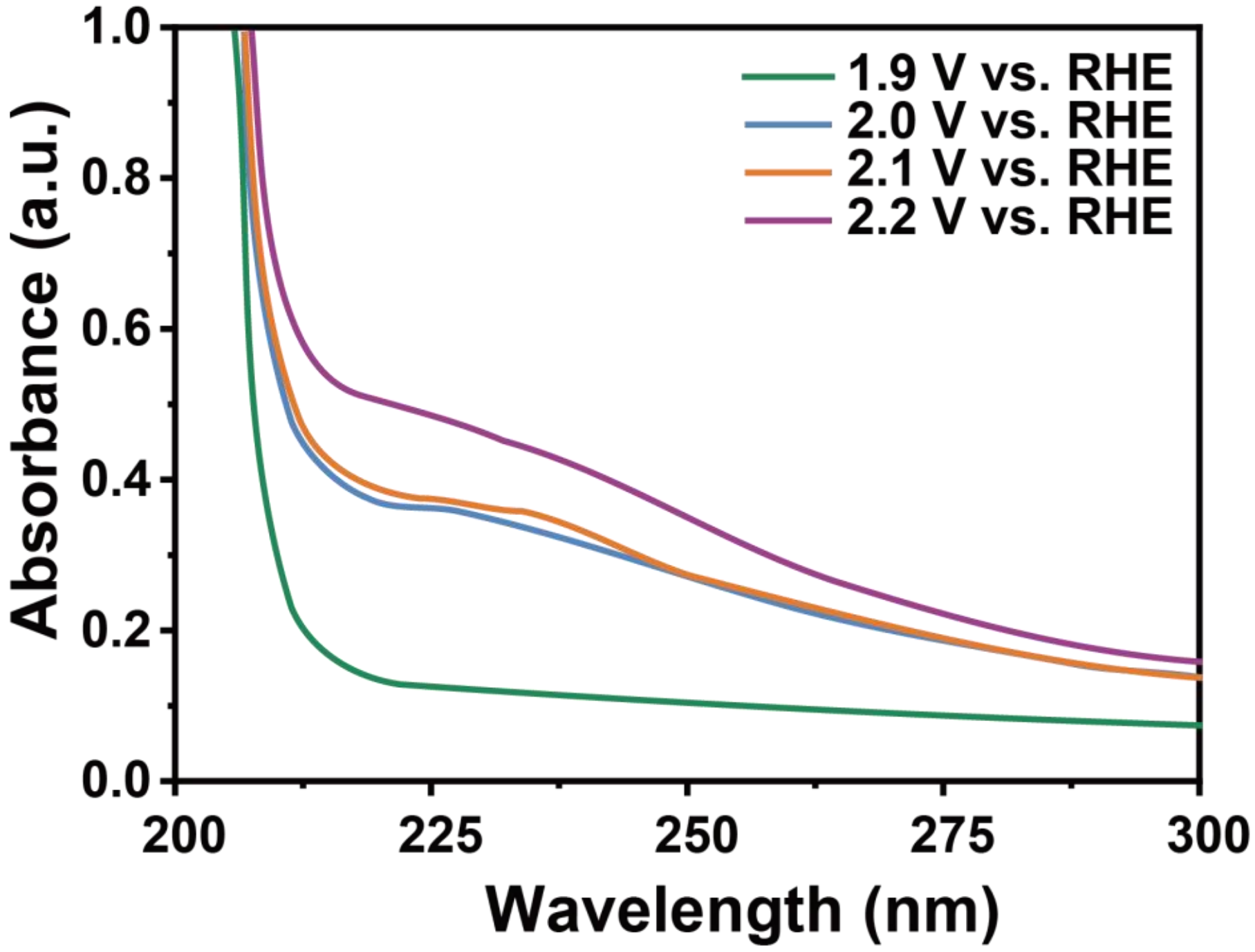

Figure S10. UV-Vis curves of electrolytes after NOR test at various potentials.

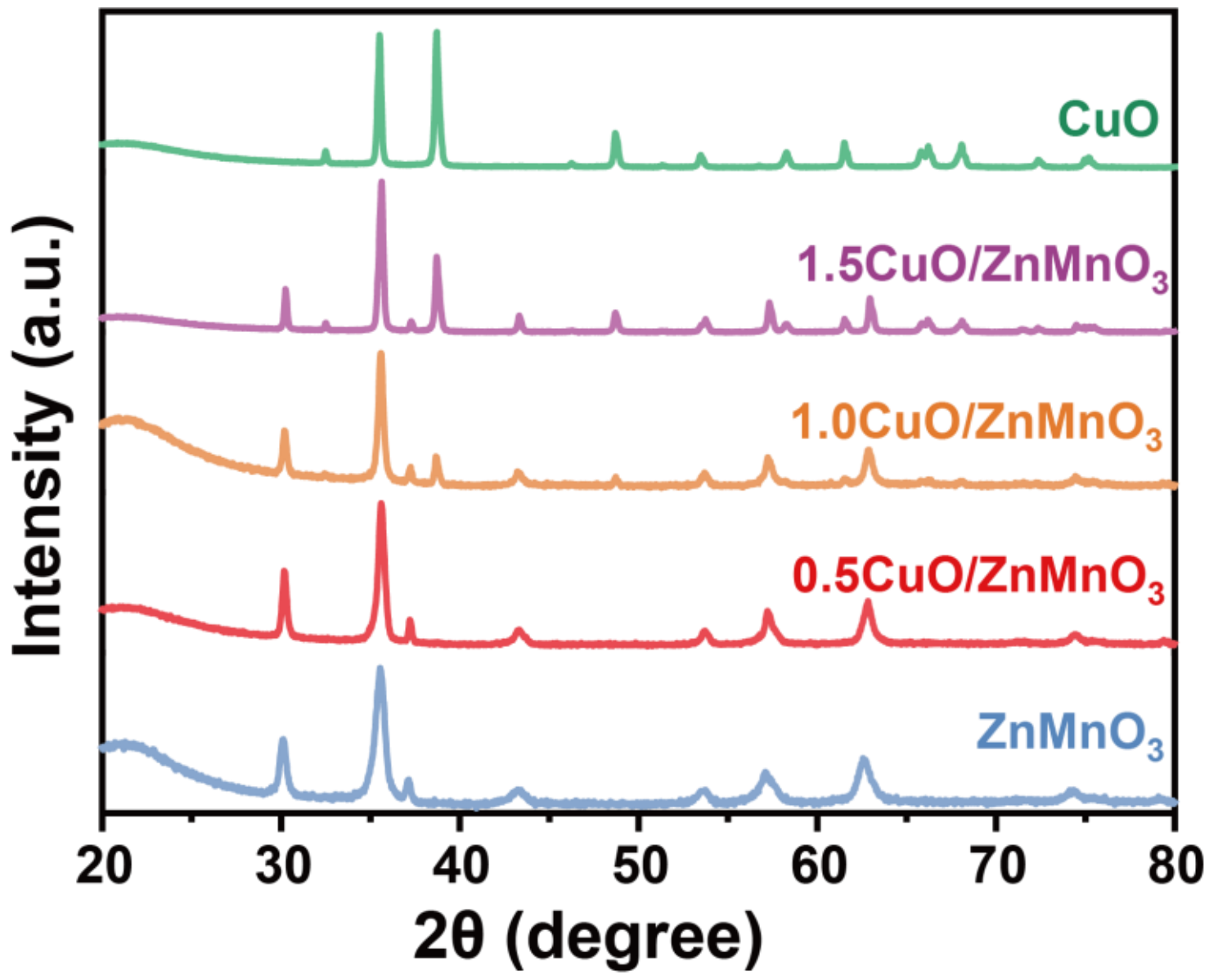

Figure S11. XRD patterns of $CuO/ZnMnO_3$ with different ratios.

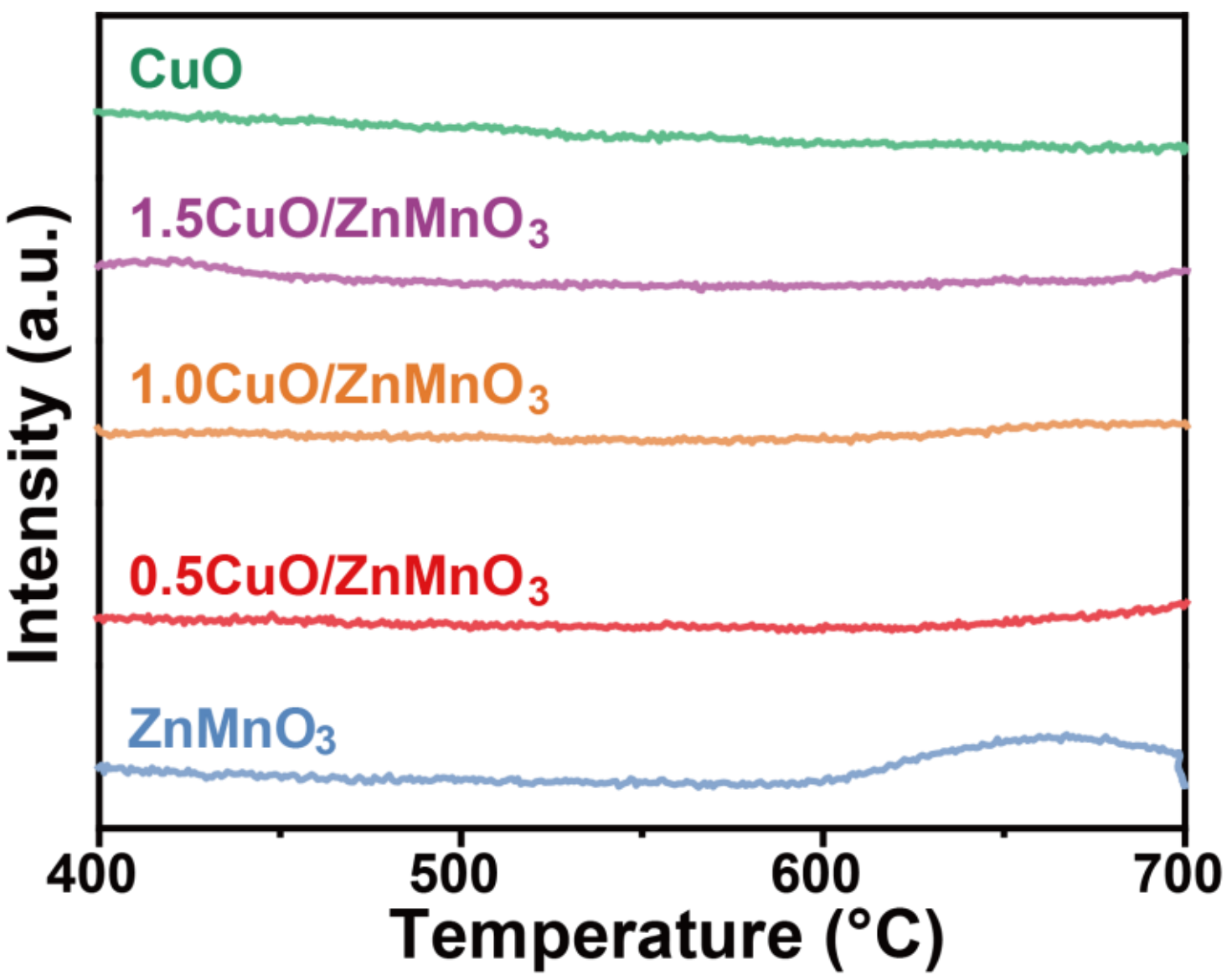

Figure S12. N_2-TPD curves of $CuO/ZnMnO_3$ with different ratios.

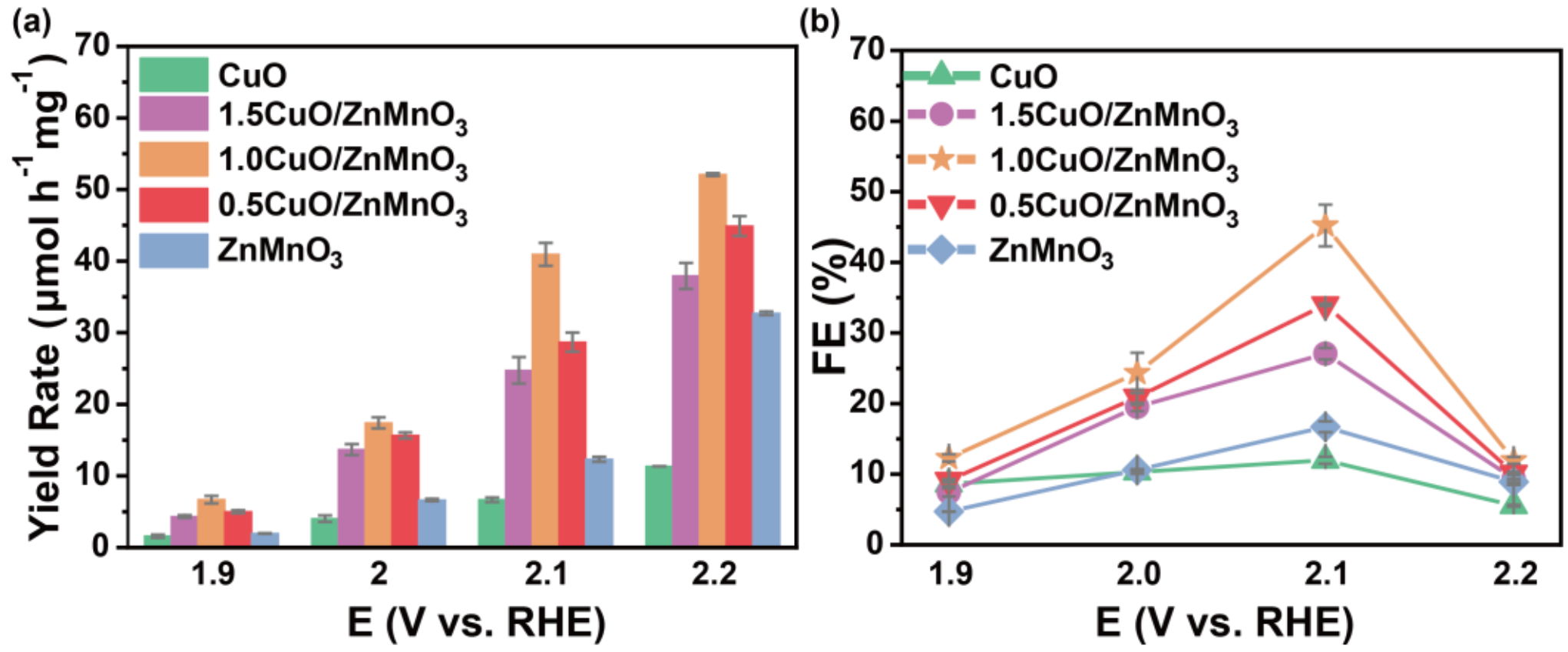

Figure S13. eNOR performance comparison of CuO/$ZnMnO_3$ heterostructure with different ratios. Nitrate yields (**a**) and FEs (**b**) of CuO/$ZnMnO_3$ heterostructure with different ratios.

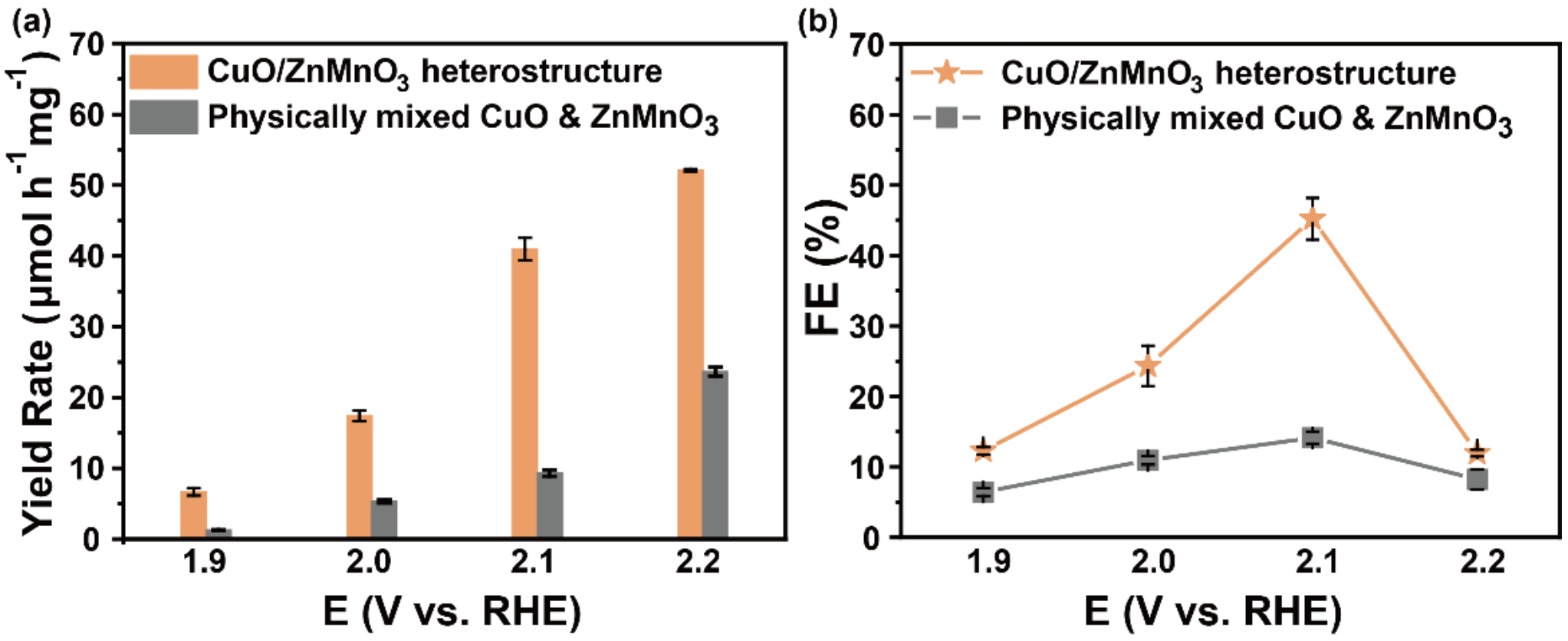

Figure S14. eNOR performance comparison between $CuO/ZnMnO_3$ heterostructure and physically mixed CuO & $ZnMnO_3$. Nitrate yields (**a**) and FEs (**b**) of $CuO/ZnMnO_3$ heterostructure and physically mixed CuO and $ZnMnO_3$ at different potentials.

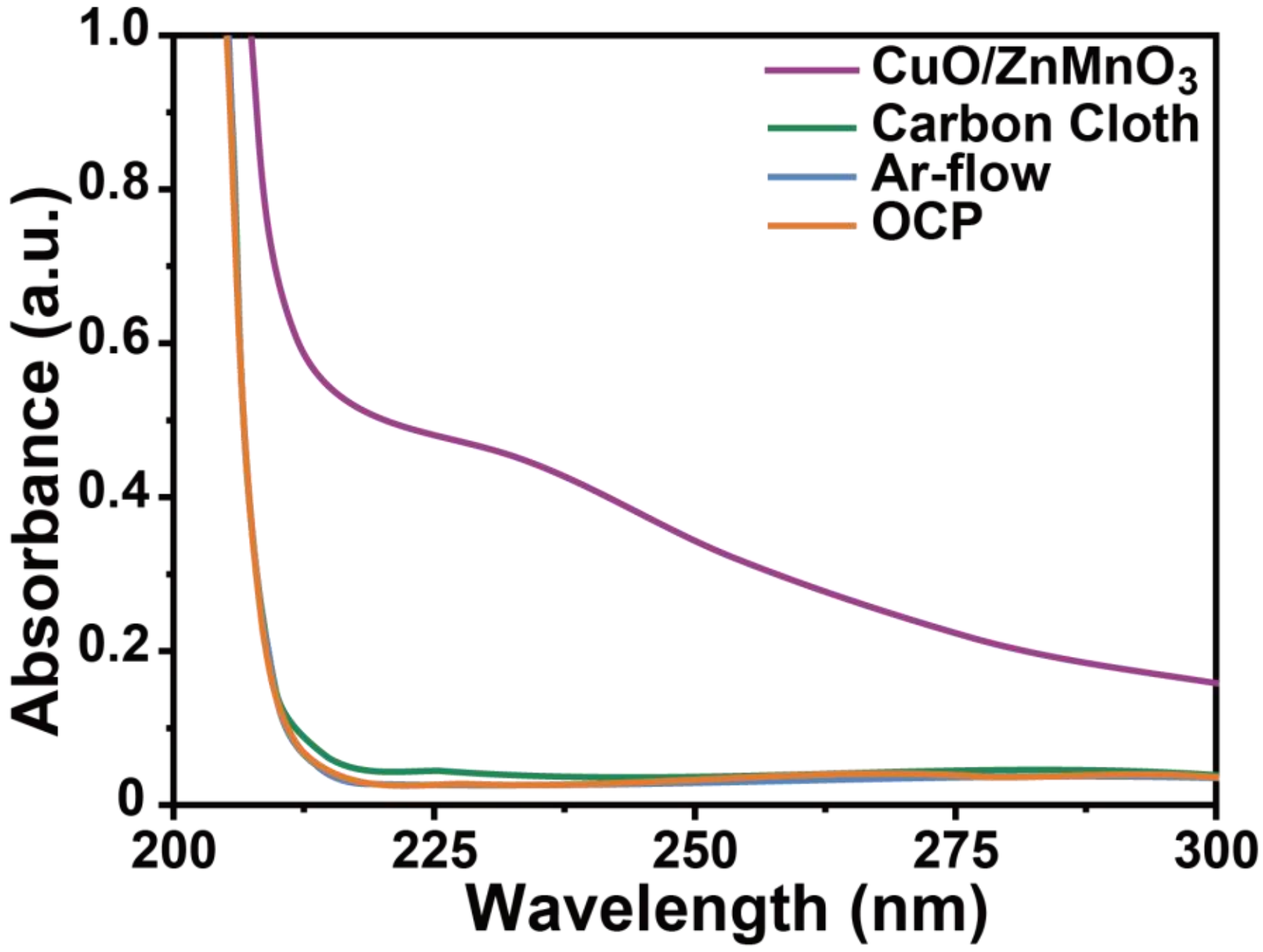

Figure S15. UV-vis absorption spectra of $CuO/ZnMnO_3$ investigated under different conditions ("O/C" represents removal of external potentials; "Ar" represents that the electrolytes are Ar-saturated) and of the bare carbon cloth (CC) electrode in N_2-saturated electrolyte.

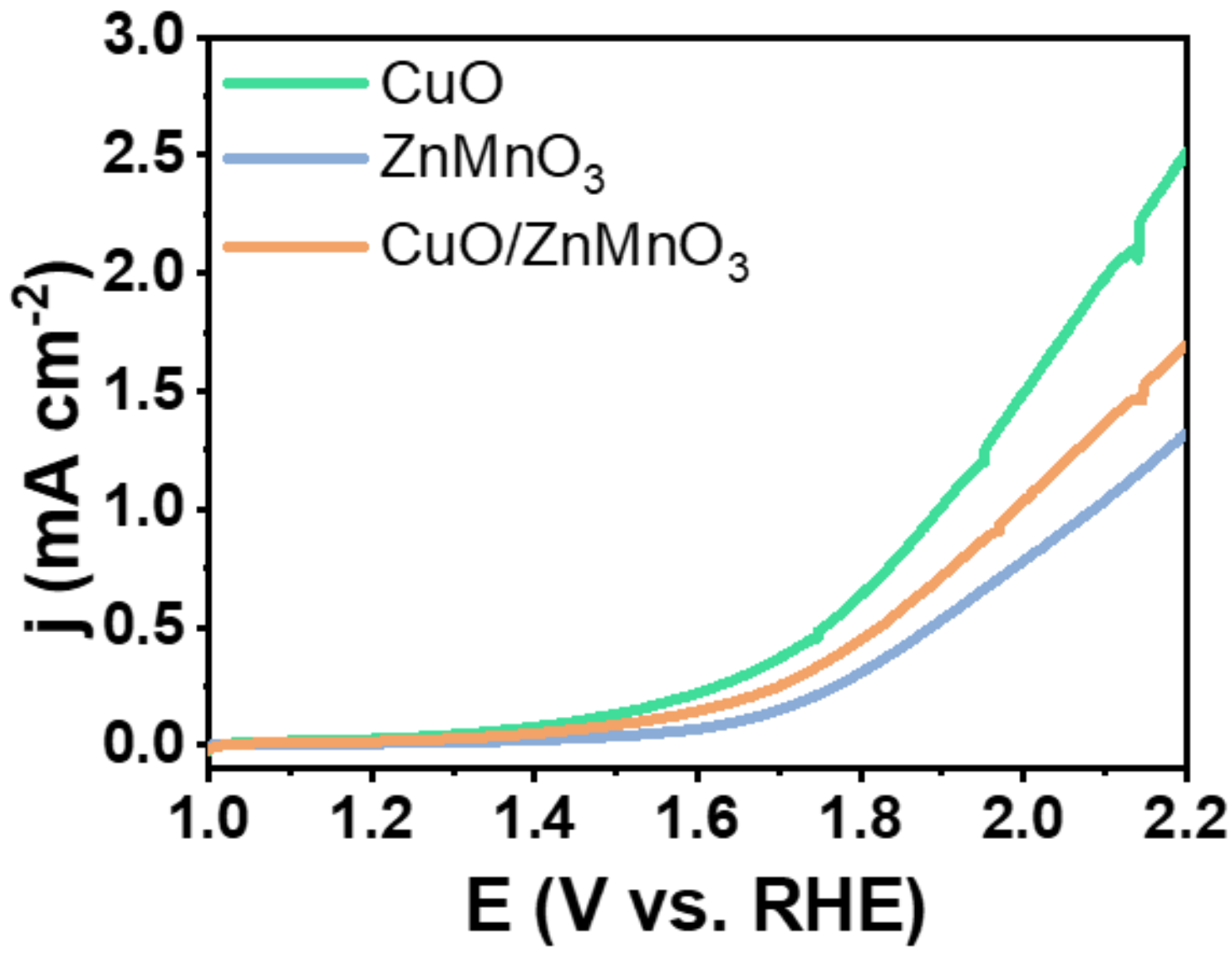

Figure S16. LSV curve of CuO, $ZnMnO_3$, and CuO/$ZnMnO_3$ in Ar-saturated 1 M KOH.

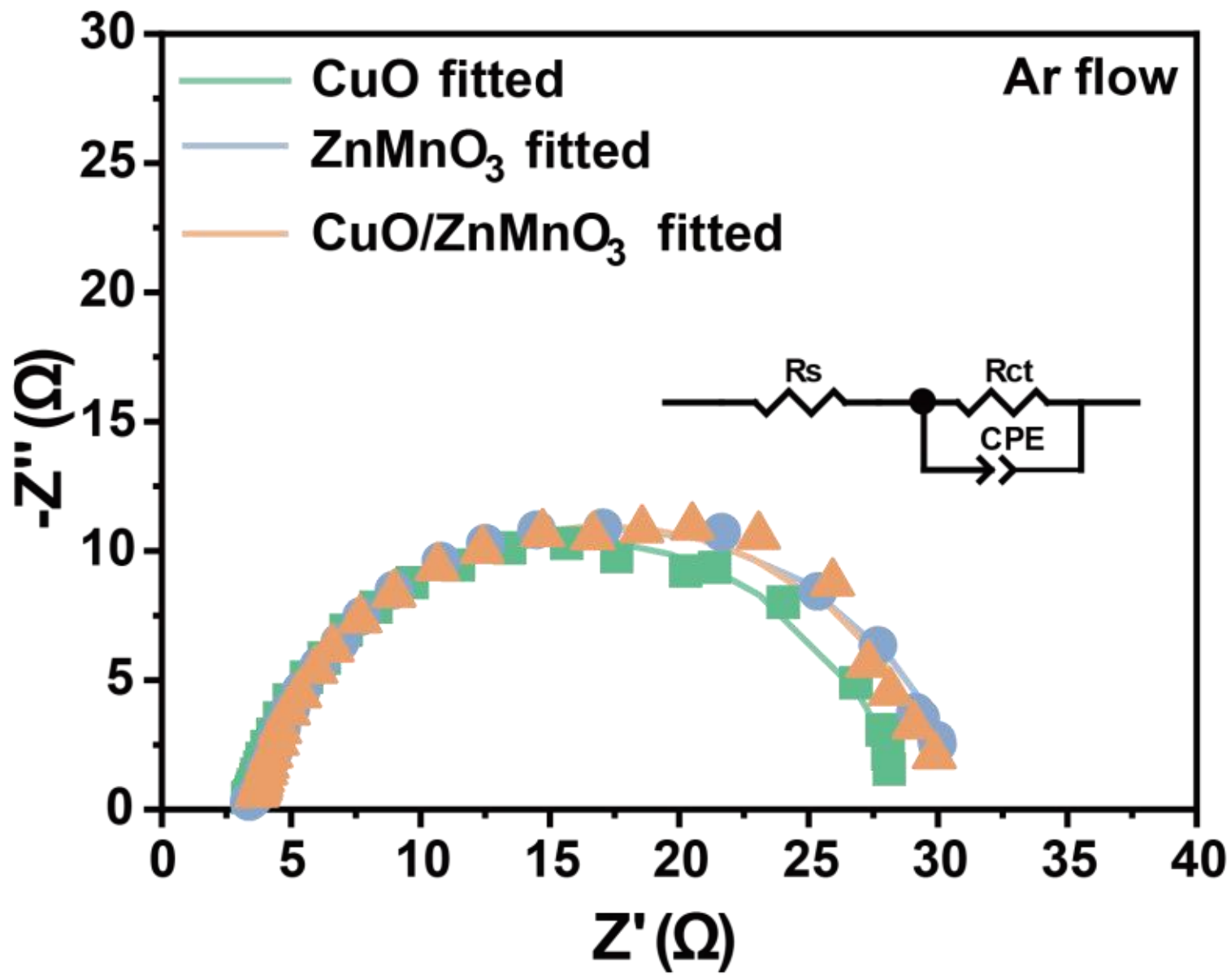

Figure S17. EIS curve of CuO, $ZnMnO_3$ and CuO/$ZnMnO_3$ under Ar flow.

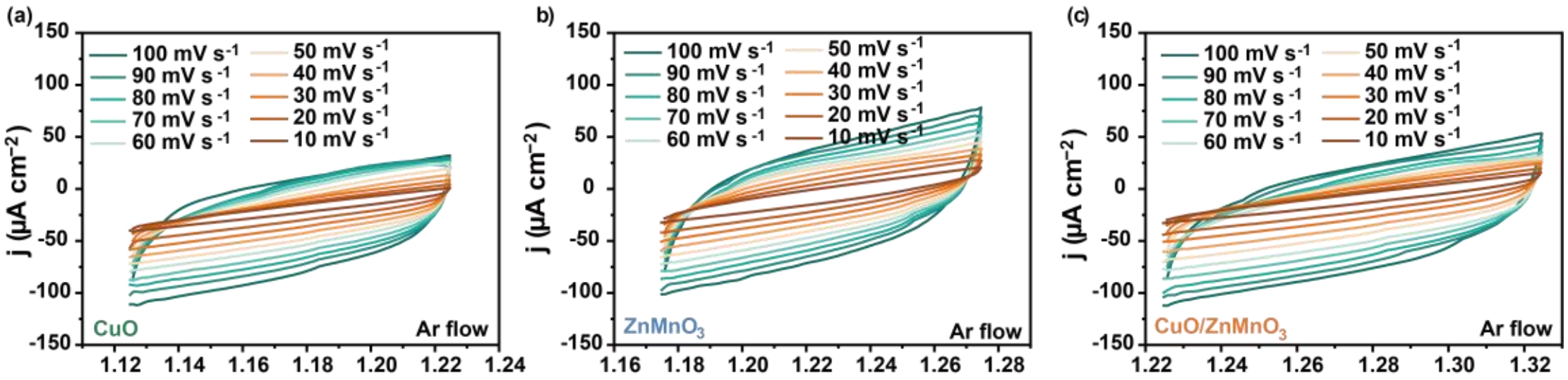

Figure S18. Corresponding CV curves at different scan rates of (**a**) CuO, (**b**) $ZnMnO_3$ and (**c**) CuO/$ZnMnO_3$ under Ar-flow.

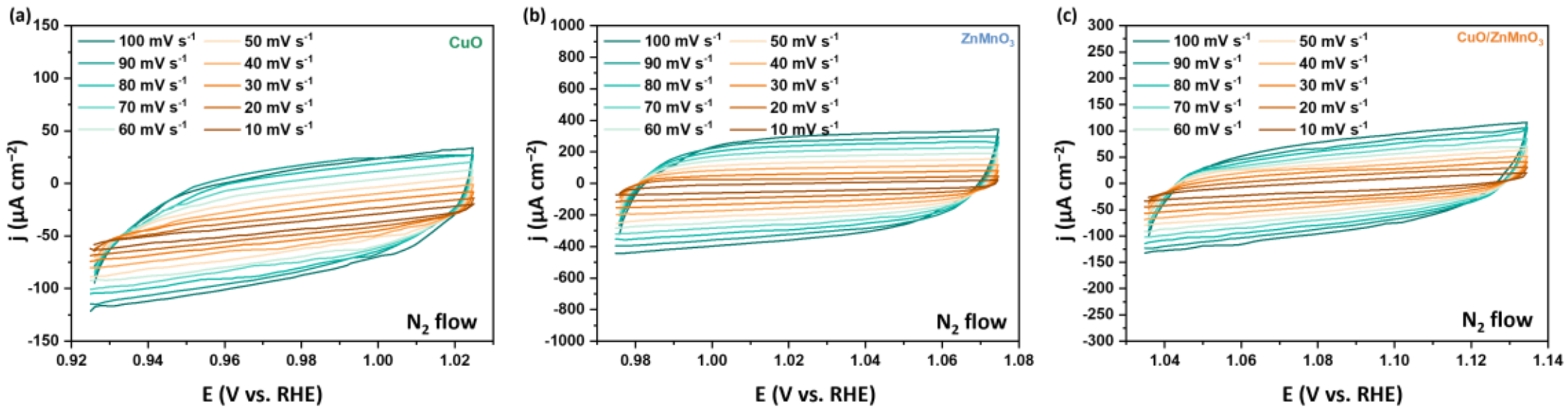

Figure S19. Corresponding CV curves at different scan rates of (**a**) CuO, (**b**) $ZnMnO_3$ and (**c**) CuO/$ZnMnO_3$ under N_2-flow.

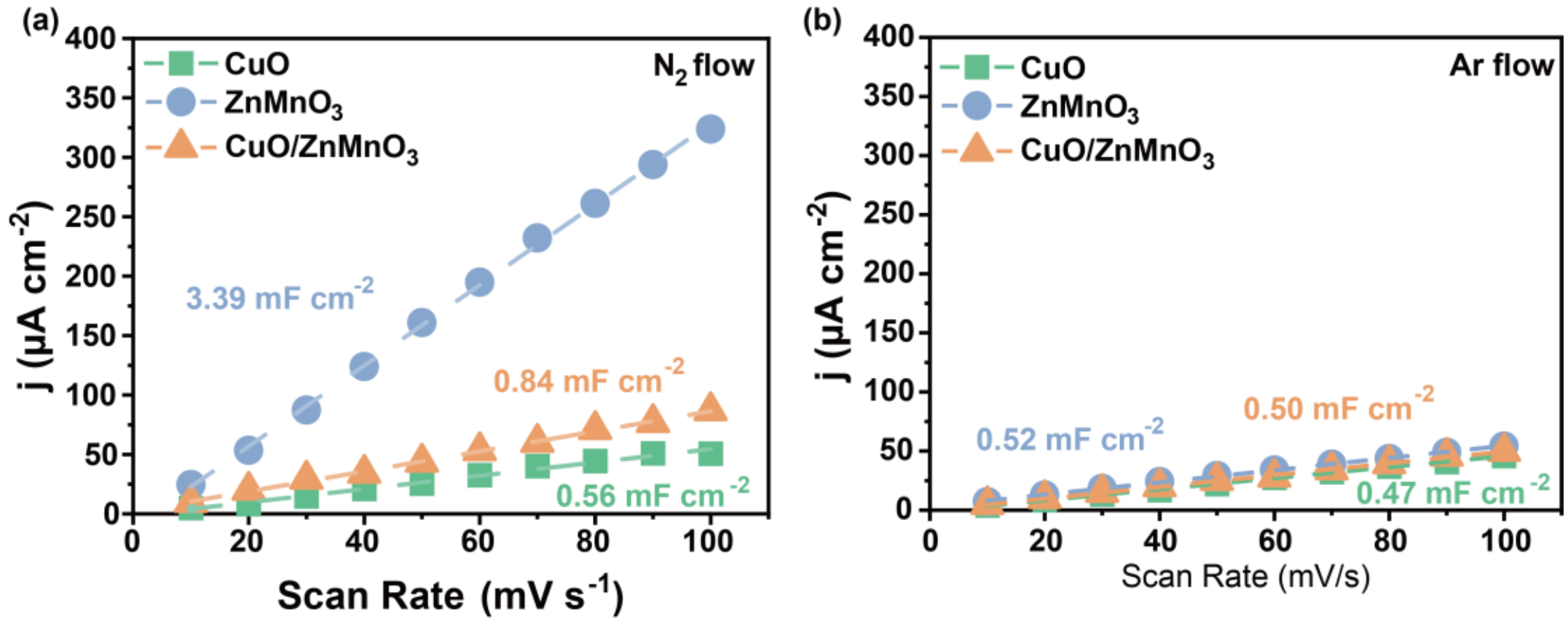

Figure S20. C_{dl} diagram of CuO, $ZnMnO_3$ and CuO/$ZnMnO_3$ under N_2 (**a**) and Ar-flow (**b**).

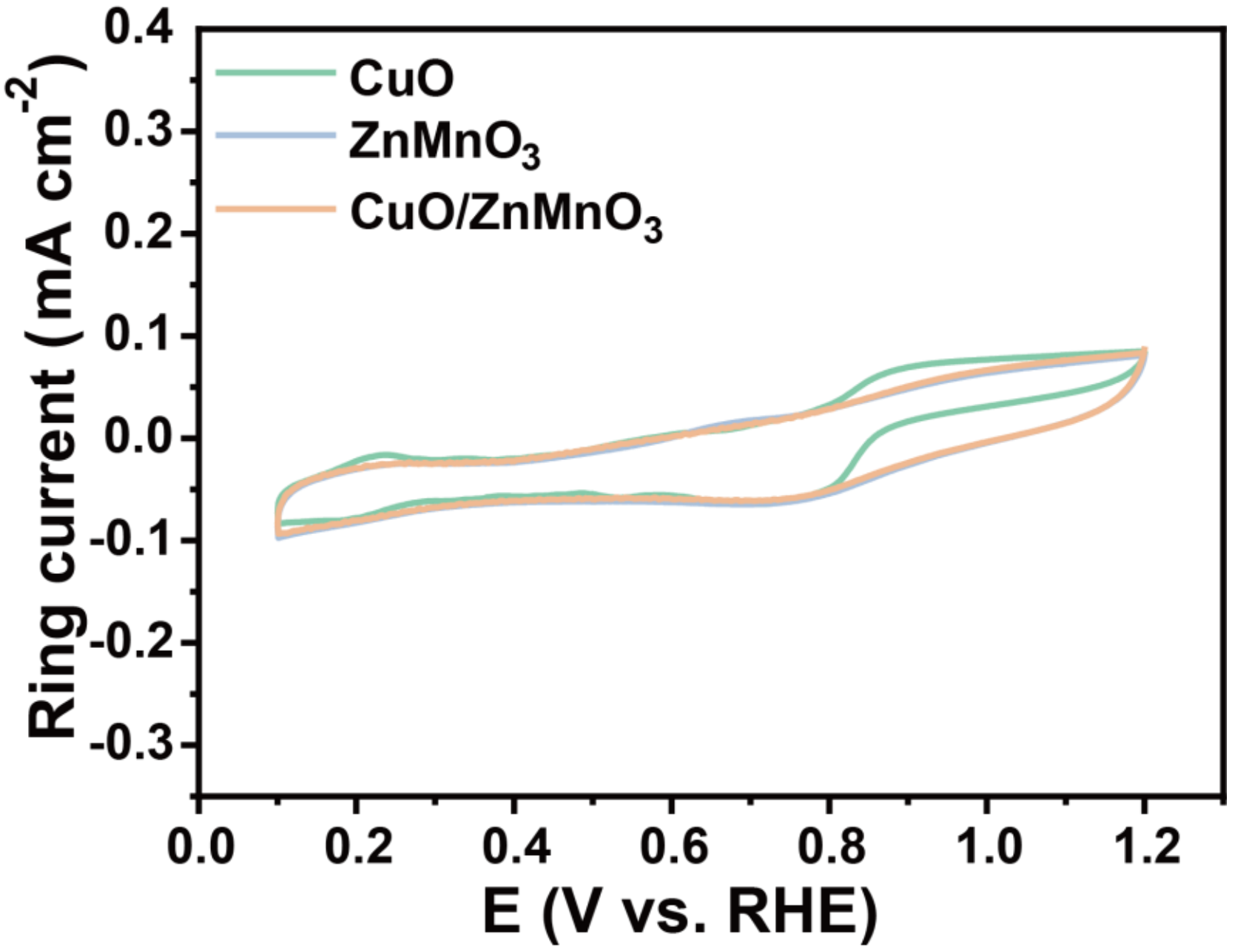

Figure S21. CVs on Pt ring; GC covered by CuO, $ZnMnO_3$ or CuO/$ZnMnO_3$ under Ar-flow. The disc electrode was fixed at 2.1V vs. RHE. RRDE rotation speed is 400 rpm.

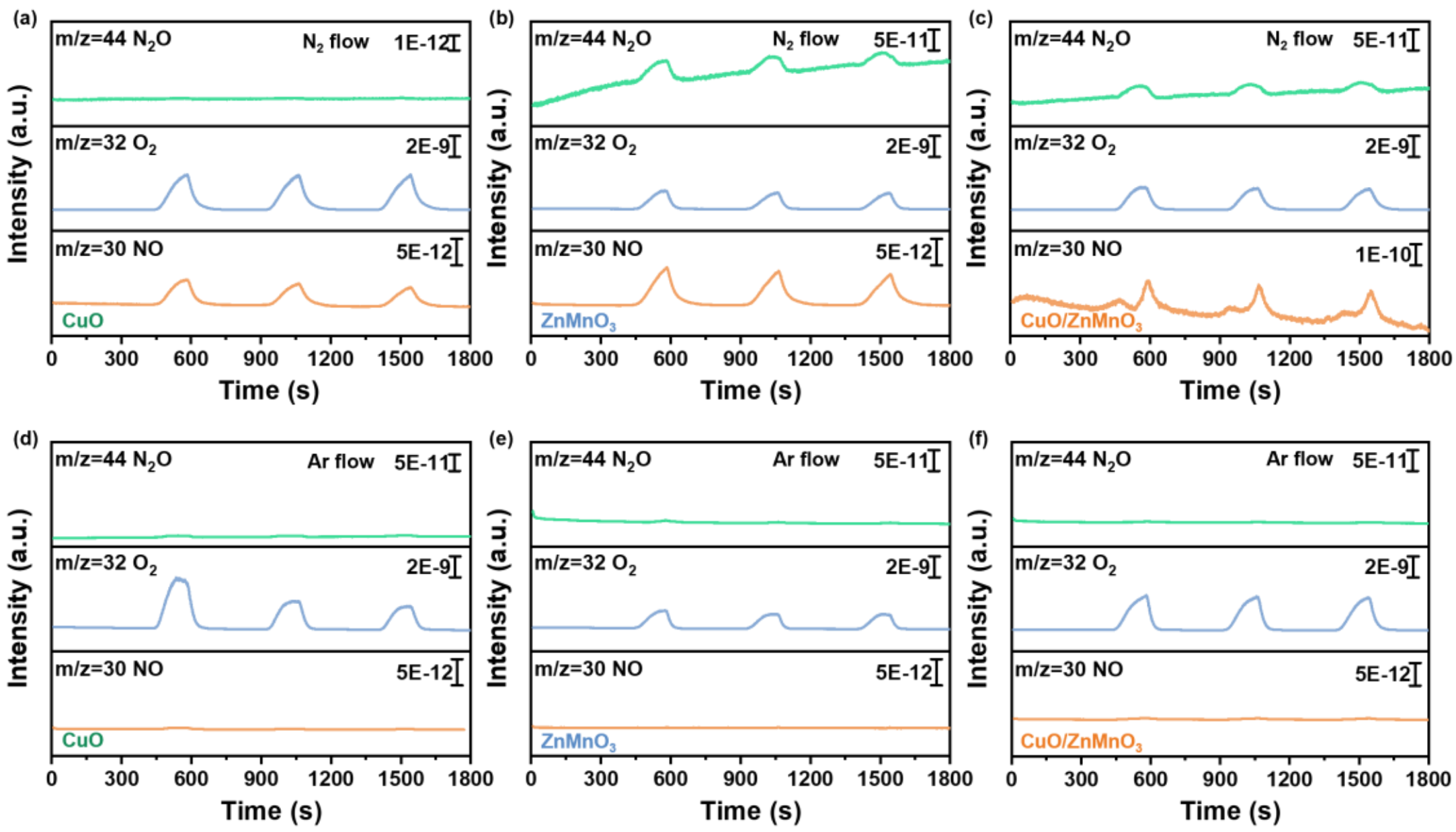

Figure S22. Online DEMS measurements of NOR over (a) CuO, (b) $ZnMnO_3$ and (c) $CuO/ZnMnO_3$ in N_2-satruated 1 M KOH electrolyte under from 1.0 V to 2.2V vs. RHE for 3 cycles. Online DEMS measurements of OER over (d) CuO, (e) $ZnMnO_3$ and (f) $CuO/ZnMnO_3$ in Ar-satruated 1 M KOH electrolyte under from 1.0 V to 2.2V vs. RHE for 3 cycles.

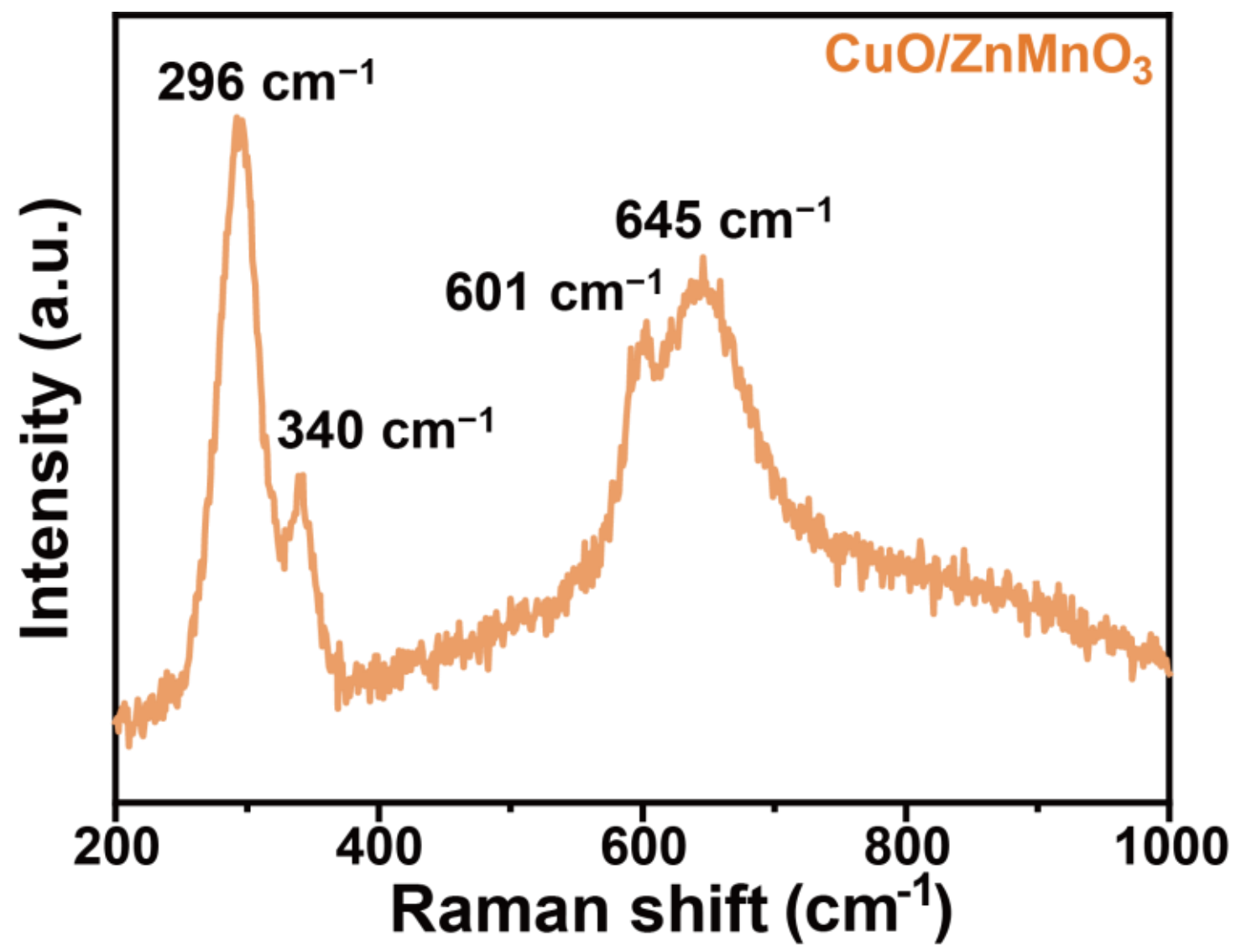

Figure S23. The Raman spectrum of $CuO/ZnMnO_3$.

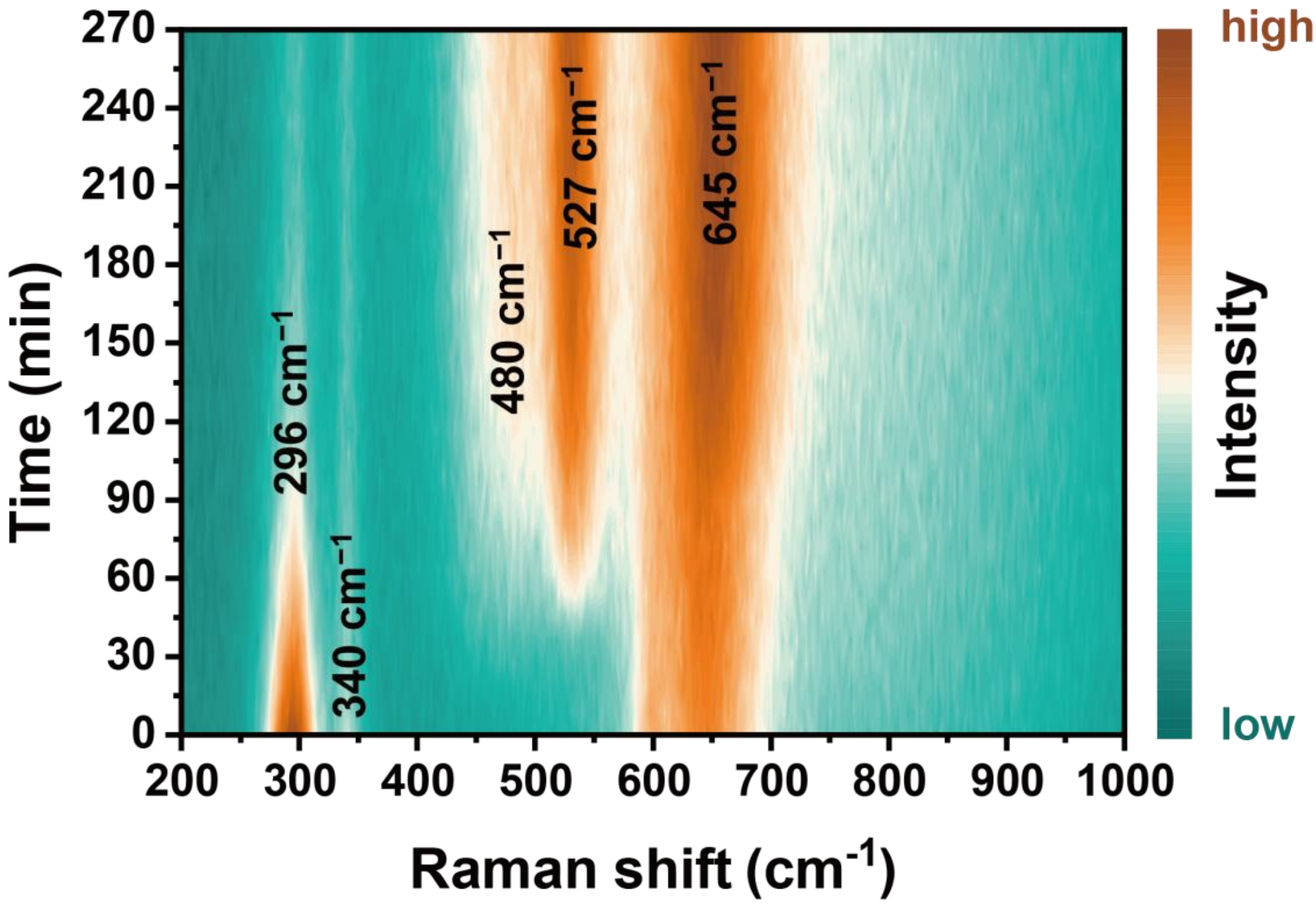

Figure S24. In-situ Raman spectrum (200 cm^{-1} – 1000cm^{-1}) of $CuO/ZnMnO_3$ under N_2-flow at 2.1 V vs. RHE in 1 M KOH for 270 min.

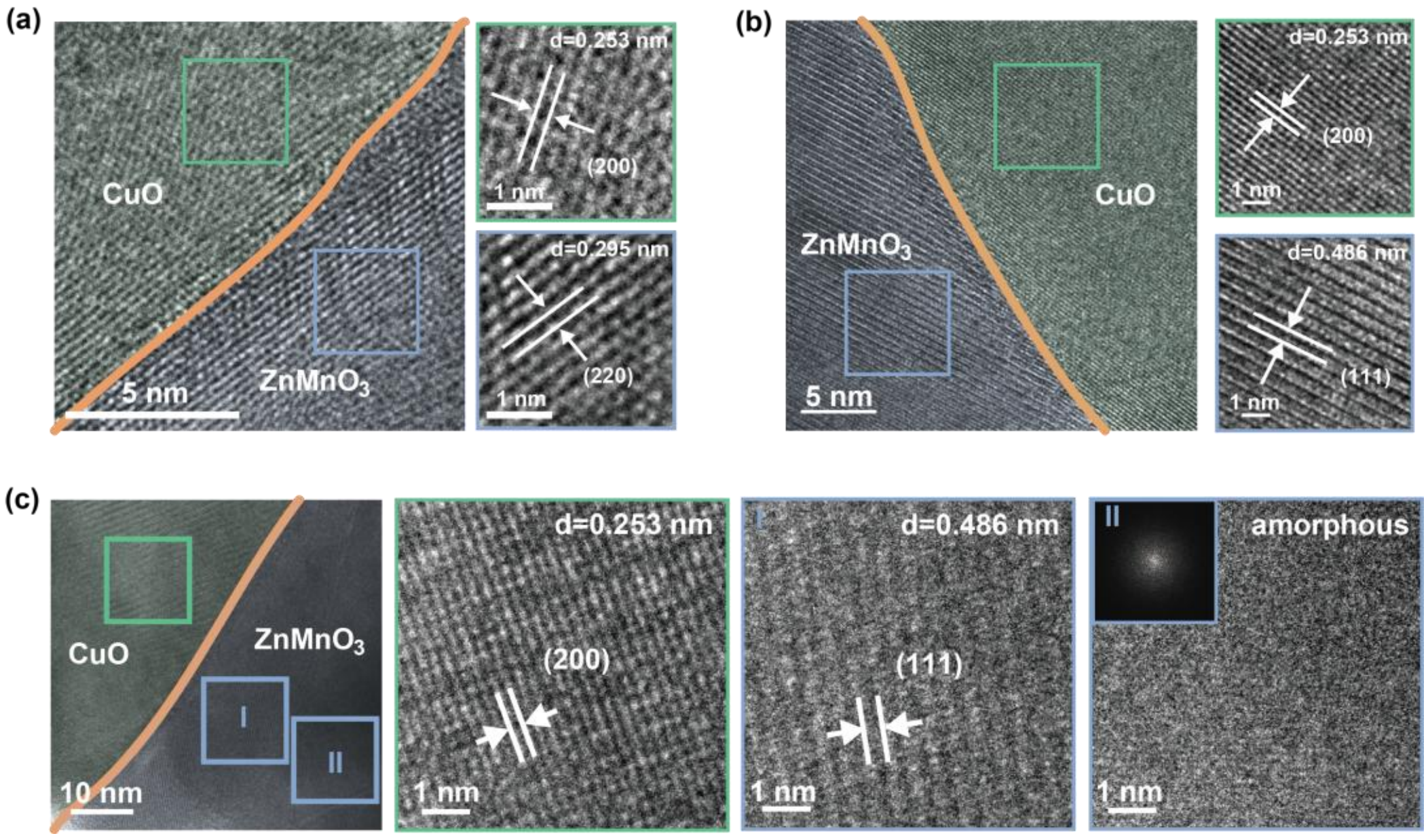

Figure S25. HRTEM image of the interface of $CuO/ZnMnO_3$ after electrocatalysis. (**a**) Interface between CuO (200) facet and $ZnMnO_3$ (220) facet. (**b**)Interface between CuO (200) facet and $ZnMnO_3$ (111) facet. (**c**) Interface between CuO (200) facet and $ZnMnO_3$ (111) facet, with partial bulk $ZnMnO_3$ phase becoming amorphous.

$$
\begin{aligned}
{}^{*}N_2 + OH^- &\rightarrow {}^{*}N_2OH + e^- \\
{}^{*}N_2OH + OH^- &\rightarrow {}^{*}N_2O + H_2O + e^- \\
{}^{*}N_2O + OH^- &\rightarrow {}^{*}N_2O_2H + e^- \\
{}^{*}N_2O_2H + OH^- &\rightarrow {}^{*}NOH + HNO_2 + e^- \\
{}^{*}NOH + OH^- &\rightarrow {}^{*}NO + H_2O + e^- \\
{}^{*}NO + OH^- &\rightarrow {}^{*}NO_2H + e^-
\end{aligned}
$$

Figure S26. Proposed nitrogen electrochemical oxidation pathway over the catalyst.

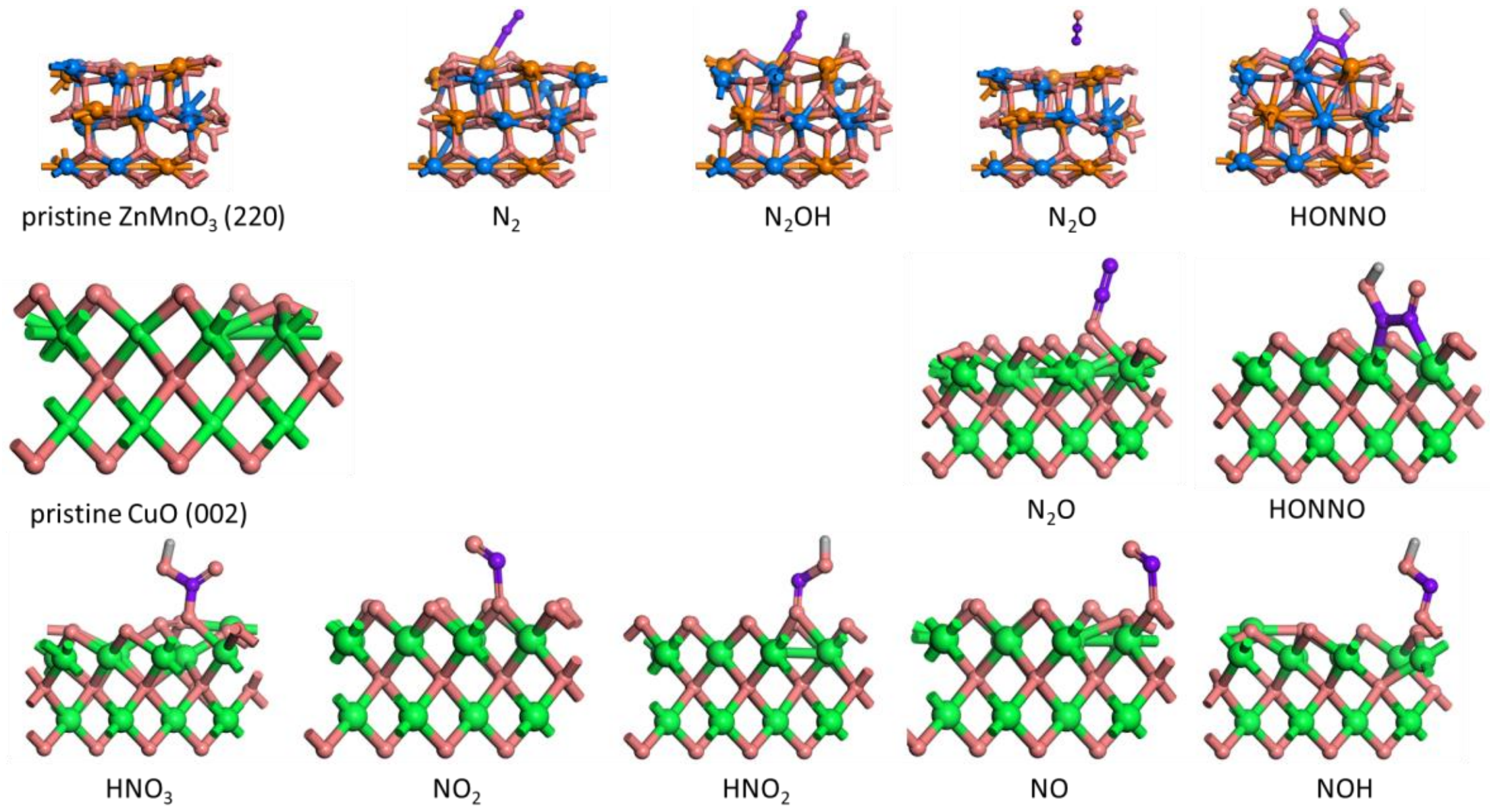

Figure S27. Simplified structures of various intermediates on the $ZnMnO_3$ (220) model and CuO (002) model along the NOR pathway.

2. Supplementary Tables

Table S1. ICP-MS results of CuO, $ZnMnO_3$ and $CuO/ZnMnO_3$.

Sample	Cu (mass%)	Zn (mass%)	Mn (mass%)
CuO	0.75	N/A	N/A
$ZnMnO_3$	N/A	0.37	0.29
$CuO/ZnMnO_3$	0.25	0.25	0.22

Table S2. XPS fitting results of Cu 2p peaks of CuO and $CuO/ZnMnO_3$.

	Position (eV)	Area	FWHM (eV)	%GL
CuO	962.2	9035.5	2.52	80
	953.8	15769.2	3.01	80
	943.7	5184.3	1.56	80
	941.5	8596.2	2.26	80
	933.8	32817.8	2.73	80
$CuO/ZnMnO_3$	961.6	1849.4	3.18	80
	953.5	2486.1	2.96	80
	942.9	1809.1	3.12	80
	940.4	728.8	1.63	80
	933.7	4382.0	2.28	80

Table S3. XPS fitting results of Mn 2p peaks of $ZnMnO_3$ and $CuO/ZnMnO_3$.

	**Position (eV)**	**Area**	**FWHM (eV)**	**%GL**
$ZnMnO_3$	642.1	8117.958	2.274	80
	653.6	6715.857	3.027	80
	640.8	1095.218	0.710	80
$CuO/ZnMnO_3$	642.1	8917.984	2.683	80
	653.7	5818.837	3.101	80
	640.8	283.939	0.700	80

Table S4. XPS fitting results of O 1s peaks of CuO, $ZnMnO_3$ and CuO/$ZnMnO_3$.

	**Position (eV)**	**Area**	**FWHM (eV)**	**%GL**
CuO	529.675	11370.370	1.093	80
	532.458	1280.349	1.249	80
	531.428	4047.371	1.408	80
$ZnMnO_3$	529.661	10197.08	0.989	80
	531.915	6704.394	1.755	80
	533.416	1886.683	1.111	80
CuO/$ZnMnO_3$	529.678	10336.05	0.982	80
	531.891	5863.419	1.768	80
	533.427	2287.014	1.257	80

Table S5. Performance comparison of $CuO/ZnMnO_3$ and other reported NOR catalysts.

**Catalyst**	**Electrolyte**	**Applied Potential (V vs. RHE)**	**Yield Rate (μmol h^{-1} cm^{-2})**	**FE**	**Anodic Energy Efficiency**
$CuO/ZnMnO_3$ This work	1 M KOH	2.1	52.1	45.2%	28.41%
$Sr_{0.9}RuO_3$[1]	0.1 M Na_2SO_4	2.2	17.9	38.60%	23.16%
$ZnFe_{0.4}Co_{1.6}O_4$[2]	1 M KOH	1.6	0.029	5.95%	4.91%
D-RuO_2[3]	0.05 M H_2SO_4	1.36	0.22	6.70%	6.50%
AD-Fe NS[4]	0.05 M K_2SO_4	2.1	2.45	35.63%	22.40%
$Pd_{0.9}Ru_{0.1}O_2$[5]	0.1 M KOH	1.7	0.039	0.40%	0.31%
Fe-SnO_2[6]	0.05 M H_2SO_4	1.96	0.069	0.84%	0.57%
Ru/$Mn_{1.04}$-RuO_2[7]	0.1 M KOH	1.4	0.85	44.29%	41.76%
Pt[8]	0.3 M K_2SO_4	2.19	0.06	1.23%	0.74%
2.79Ru/TiO_2[9]	0.1 M Na_2SO_4	1.8	0.016	26.10%	19.14%
TiO_{2-x}/Cu-NWs[10]	0.1 M KOH	1.56	0.69	22.04%	18.65%
HOCo/GDY[11]	1 M KOH	1.6	3.05	21.5%	17.74%
Ru–Mn_3O_4[12]	0.1 M Na_2SO_4	2	0.078	28.87%	19.05%
Pd-MX[13]	0.01 M Na_2SO_4	2.03	0.007	11.34%	7.37%

3. References

(1) Zheng, H.; Ma, Z.; Liu, Y.; Zhang, Y.; Ye, J.; Debroye, E.; Zhang, L.; Liu, T.; Xie, Y. Perovskite oxide as a new platform for efficient electrocatalytic nitrogen oxidation. *Angew. Chem. Int. Ed.* **2024**, *63* (1), e202316097. DOI: 10.1002/anie.202316097

(2) Dai, C.; Sun, Y.; Chen, G.; Fisher, A. C.; Xu, Z. J. Electrochemical Oxidation of Nitrogen towards Direct Nitrate Production on Spinel Oxides. *Angew. Chem. Int. Ed.* **2020**, *59* (24), 9418-9422. DOI: 10.1002/anie.202002923

(3) Xu, F.; Liu, X.; Zhang, L.; Guo, M.; Li, M.; Ding, X.; Zhang, L. Revealing and optimizing the dialectical relationship between NOR and OER: cation vacancy engineering enables RuO_2 with unanticipated high electrochemical nitrogen oxidation performance. *Advanced Energy Materials* **2023**, *13* (22), 2300615. DOI: 10.1002/aenm.202300615

(4) Guo, Y.; Zhang, S.; Zhang, R.; Wang, D.; Zhu, D.; Wang, X.; Xiao, D.; Li, N.; Zhao, Y.; Huang, Z.; et al. Electrochemical Nitrate Production via Nitrogen Oxidation with Atomically Dispersed Fe on N-Doped Carbon Nanosheets. *ACS Nano* **2022**, *16* (1), 655-663. DOI: 10.1021/acsnano.1c08109

(5) Li, T.; Han, S.; Wang, C.; Huang, Y.; Wang, Y.; Yu, Y.; Zhang, B. Ru-Doped Pd Nanoparticles for Nitrogen Electrooxidation to Nitrate. *ACS Catalysis* **2021**, *11* (22), 14032-14037. DOI: 10.1021/acscatal.1c04360

(6) Zhang, L.; Cong, M.; Ding, X.; Jin, Y.; Xu, F.; Wang, Y.; Chen, L.; Zhang, L. A Janus Fe-SnO_2 Catalyst that Enables Bifunctional Electrochemical Nitrogen Fixation. *Angew. Chem. Int. Ed.* **2020**, *59* (27), 10888-10893. DOI: 10.1002/anie.202003518

(7) Zeng, H.; Wang, D.; Yang, C.-J.; Dong, C.-L.; Lin, W.; Sang, X.; Yang, B.; Li, Z.; Yao, S.; Zhang, Q.; et al. Interfacial Electron Engineering Unlocks Efficient Nitrate

Electrosynthesis by Balancing Nitrogen Activation and Oxygen Evolution. *ACS Catalysis* **2025**, *15* (11), 9610-9620. DOI: 10.1021/acscatal.5c01568

(8) Wang, Y.; Li, T.; Yu, Y.; Zhang, B. Electrochemical synthesis of nitric acid from nitrogen oxidation. *Angew. Chem. Int. Ed.* **2022**, *61* (12), e202115409. DOI: 10.1002/anie.202115409

(9) Kuang, M.; Wang, Y.; Fang, W.; Tan, H.; Chen, M.; Yao, J.; Liu, C.; Xu, J.; Zhou, K.; Yan, Q. Efficient Nitrate Synthesis via Ambient Nitrogen Oxidation with Ru-Doped TiO_2/RuO_2 Electrocatalysts. *Adv. Mater.* **2020**, *32* (26), 2002189. DOI: 10.1002/adma.202002189

(10) Li, Q.; Xiao, Z.; Jia, W.; Li, Q.; Li, X.; Wang, W. Copper nanowires decorated with TiO_{2-x} from MXene for enhanced electrocatalytic nitrogen oxidation into nitrate under vacuum assistance. *Nano Research* **2023**, *16* (10), 12357-12362. DOI: 10.1007/s12274-023-6126-8

(11) Zheng, X.; Wu, H.; Gao, Y.; Chen, S.; Xue, Y.; Li, Y. Controllable assembly of highly oxidized cobalt on graphdiyne surface for efficient conversion of nitrogen into nitric acid. *Angew. Chem. Int. Ed.* **2024**, *63* (9), e202316723. DOI: 10.1002/anie.202316723

(12) Nie, Z.; Zhang, L.; Ding, X.; Cong, M.; Xu, F.; Ma, L.; Guo, M.; Li, M.; Zhang, L. Catalytic Kinetics Regulation for Enhanced Electrochemical Nitrogen Oxidation by Ru-Nanoclusters-Coupled Mn_3O_4 Catalysts Decorated with Atomically Dispersed Ru Atoms. *Adv. Mater.* **2022**, *34* (14), 2108180. DOI: 10.1002/adma.202108180

(13) Fang, W.; Du, C.; Kuang, M.; Chen, M.; Huang, W.; Ren, H.; Xu, J.; Feldhoff, A.; Yan, Q. Boosting efficient ambient nitrogen oxidation by a well-dispersed Pd on MXene electrocatalyst. *Chem. Commun.* **2020**, *56* (43), 5779-5782. DOI: 10.1039/D0CC01759K
